# Emergency care competencies for non–emergency medicine specialists in Japan: a modified Delphi study

**DOI:** 10.1186/s12909-026-10094-0

**Published:** 2026-08-08

**Authors:** Atsushi Jinno, Shuichi Miyamoto, Kazuhito Nomura, Hiroshi Mihara, Masanori Shiratori, Yoshihisa Tsuji

**Affiliations:** 1 Department of General Medicine, Sapporo Medical University, Sapporo, Hokkaido Japan; 2 Minamihiyama Regional Medical Education Course, Sapporo Medical University, Sapporo, Hokkaido Japan; 3 Department of General Medicine, Shiga University of Medical Science, Otsu, Shiga Japan

**Keywords:** Clinical competence, Delphi technique, Emergency medicine, Curriculum, Non-specialist physicians, Japan

## Abstract

**Background:**

Physicians from specialties other than emergency medicine (non-EM specialists) provide first-contact emergency care in many health systems. Existing emergency care courses and competency frameworks address resource-limited first-contact care or rural self-sufficient practice, but do not define the role-specific competencies expected of non-EM specialists in high-resource, specialist-limited settings. This study aimed to develop a consensus-based set of core emergency care competencies for non-EM specialists in Japan.

**Methods:**

We conducted a modified Delphi consensus study from January to March 2026. A balanced national panel of 50 specialists, comprising 25 EM and 25 non-EM specialists, rated emergency care items on a 7-point Likert scale. Items were adopted if they met all predefined criteria: median score ≥ 6, ≥ 80% of ratings in the 6–7 range, and quartile deviation ≤ 1.0. Items were classified as consensus non-adoptions if median score ≤ 3, ≥ 70% of ratings in the 1–3 range, and quartile deviation ≤ 2.0, or as non-adopted by persistent lack of consensus if they met neither set of criteria across two consecutive rounds. Items were generated from a structured review of training documents and iteratively revised using panellist feedback.

**Results:**

Of the 50 enrolled panellists, 48, 42, and 41 completed Rounds 1, 2, and 3, respectively. From an initial pool of 121 items, 38 reached adoption consensus: 23 in Round 1 and 15 in Round 2. Adopted competencies clustered around recognition of acute deterioration, interpretation of laboratory and imaging findings, initial stabilisation, judgement of indications for specialist-level intervention, and timely escalation. Several high-risk invasive procedures, including cricothyrotomy, chest tube insertion, pericardiocentesis, and intubation for non-arrest patients, did not reach consensus as universal core competencies for non-EM specialists.

**Conclusions:**

Expert consensus defined a distinct, role-specific competency profile for non-EM specialists in Japan, weighted towards recognition, diagnostic interpretation, clinical judgement, initial stabilisation, and escalation rather than a reduced version of EM specialist training. The resulting 38 competencies provide content validity evidence for a minimum core of emergency care education aligned with the role non-EM specialists are expected to perform.

**Supplementary Information:**

The online version contains supplementary material available at https://doi.org/10.1186/s12909-026-10094-0.

## Background

Physicians without emergency medicine (EM) specialty training routinely provide first-contact emergency care in a range of health systems [1, 2]. This role is often discussed in rural, remote, or resource-limited settings, yet non-EM physicians also provide emergency care in well-resourced settings where EM specialist coverage is uneven. Workforce arrangements differ across countries, but training programmes face a common challenge: defining and developing the emergency care competencies these physicians need for their expected clinical roles.

Several established frameworks address emergency care outside EM specialist practice. The World Health Organization Basic Emergency Care framework targets first-contact care in resource-limited environments [3], and rural generalist curricula, including Australian emergency medicine training pathways, prepare physicians for relatively autonomous practice in geographically isolated settings [4, 5]. Both define roles for physicians working where specialist support is largely absent.

However, these frameworks do not define the core competencies expected of non-EM physicians who practise in diagnostically enabled settings, where laboratory testing, imaging, and inpatient services are available but EM specialist support is inconsistent. In such settings, the boundary between universal core competencies and advanced or context-dependent ones remains unclear. Clarifying this boundary is necessary to align education with the roles these physicians are expected to perform.

Japan exemplifies this configuration. Many secondary emergency facilities and after-hours services rely on physicians from non-EM specialties for first-contact emergency care, even though diagnostic resources such as emergency laboratory testing and computed tomography are widely available [6], because EM specialists remain concentrated in tertiary centres [7, 8]. Japan is therefore a diagnostically enabled but specialist-limited setting in which the competencies expected of non-EM specialists require clarification.

This study aimed to develop, through expert consensus, a set of role-specific emergency care competencies for non-EM specialists providing first-contact emergency care in Japan's diagnostically enabled but specialist-limited setting.

## Methods

### Study design and ethical considerations

We conducted a modified Delphi study to develop a consensus-based set of core emergency care competencies for non-EM specialists in Japan. The study design and reporting were guided by the Conducting and REporting of DElphi Studies (CREDES) recommendations [9] (Supplementary Table S1). A modified Delphi design with a predefined item pool was chosen to enable structured, anonymous consensus development on concrete clinical actions and decisions [10]. The process ran from January to March 2026, following item development and questionnaire pre-testing in July 2025.

This study was conducted in accordance with the Declaration of Helsinki and the Ethical Guidelines for Medical and Biological Research Involving Human Subjects (Japan). This study was approved by the Institutional Review Board of Sapporo Medical University (approval no. 7–1–41). All panellists provided electronic informed consent before participating in Round 1.

### Selection of experts

We used a purposive panel design with equal numbers of EM and non-EM specialists. The two groups were balanced because defining these competencies required both the safety and role-relevance judgement of EM specialists and experiential judgement of physicians who provide emergency care without EM certification. We set an enrolment target of 50 panellists, 25 per group, to allow for attrition while maintaining an adequate final panel size.

All panellists were required to be physicians licensed in Japan, hold a relevant specialty board certification, have at least three years of emergency care experience in Japan, and report managing at least 100 emergency cases annually. EM specialists were defined as physicians certified by the Japanese Association for Acute Medicine (JAAM). Non-EM specialists were defined as board-certified physicians whose primary specialty was not emergency medicine, who did not hold JAAM certification, and who provided first-contact emergency care in hospital-based emergency departments or after-hours emergency settings.

We recruited panellists between 17 and 30 November 2025 through three Japanese physician mailing lists covering emergency care, hospital medicine, and general medicine, supplemented by snowball sampling. Recruitment was stratified by expertise group. When eligible applicants exceeded the target in either group, the research team selected panellists by geographic region, hospital type, type of emergency facility, primary specialty, annual emergency case volume, years of emergency care experience, and experience in geographically isolated or underserved settings, to avoid overrepresentation of any single clinical context.

### Delphi rounds

The Delphi process was conducted online. Each round was open for approximately two weeks, followed by an analysis and feedback preparation period of approximately ten days, with two automated reminders sent during each response period. All rounds used a 7-point Likert scale (1 = strongly disagree, 7 = strongly agree).

The initial item pool was developed specifically for this study. The research team generated the item pool through a structured review of four primary sources, supplemented by iterative discussion among the investigators: (1) the JAAM Specialist Training Behavioural Objectives [11]; (2) the JAAM position paper on the "Japanese-style ER" [12]; (3) the Japan Primary Care Association New Family Medicine Specialist Training Programme [13]; and (4) simulation-based curriculum content for emergency medicine residents [14]. Candidate skills and clinical decisions were extracted, and overlapping, overly broad, or context-specific elements were consolidated, excluded, or rephrased through research team discussion. Each item was drafted as a discrete clinical skill or decision, phrased as a single observable action. The draft questionnaire was pre-tested in July 2025 with nine physicians—two EM and seven non-EM specialists—who were not enrolled in the main Delphi panel, and minor wording revisions were made on the basis of feedback regarding item clarity, response burden, and online survey operation. The resulting initial pool comprised 121 items (Supplementary Table S2).

Consensus criteria were predefined before Round 1, drawing on previous modified Delphi studies in medical education and related consensus methods [15, 16]. An item was adopted if it met all three of the following: median ≥ 6, ≥ 80% of ratings in the 6–7 range, and quartile deviation (QD) ≤ 1.0. An item was classified as a consensus non-adoption if it met all three of: median ≤ 3, ≥ 70% of ratings in the 1–3 range, and QD ≤ 2.0. Items meeting neither set were carried forward with anonymised feedback; items that met neither the adoption nor the consensus non-adoption criteria across two consecutive rounds were classified as non-adopted by persistent lack of consensus. The adoption criteria were intentionally stringent because adopted items were intended to represent core competencies expected of non-EM specialists across general emergency care contexts.

In Round 1, panellists rated all 121 items and could propose new items or suggest rewording through free-text fields. They were given three framing instructions. First, they were asked not to assume any specific institutional level. Second, they were asked to rate whether each skill should be acquired by non-EM specialists to a level at which they could perform it independently, defined as performing the skill without relying on another medical specialist, while assuming routine clinical support such as nursing assistance. Third, they were asked to rate each item as a normative general standard rather than according to current practice at their own institution. These instructions were intended to keep the diagnostically enabled but specialist-limited Japanese setting as the shared frame of reference while removing anchoring to any individual institution.

Before Round 2, the research team reviewed all items not resolved in Round 1 together with panellists' free-text comments. Items requiring only minor wording changes were retained as continuing items, whereas items that were substantively reworded, split into more specific clinical actions, or newly proposed were treated as new items in Round 2.

For Round 2, panellists received anonymised group-level feedback for each item—response distribution, median, QD, the proportions of responses in the 1–3 and 6–7 ranges, and their own previous response where applicable—and were asked to re-rate each remaining item.

Round 3 used the same anonymised feedback structure and framing instruction. No new items were introduced after Round 2. The Delphi process was stopped after Round 3 because all remaining items could be classified according to the predefined criteria.

Following Round 3, the research team grouped the adopted items post hoc into clinically meaningful categories to facilitate presentation and interpretation. This grouping was not subjected to Delphi rating or consensus; the adopted items, not the category structure, therefore constitute the consensus-derived output.

### Statistical analysis and software

For each item in each round, we computed the response distribution, median, QD, and proportions of responses in the 1–3 and 6–7 ranges, using the number of panellists who rated that item in that round as the denominator. Item classification was based on ratings from the full panel; subgroup results were not used for consensus classification. We conducted descriptive subgroup analyses by calculating median ratings separately for the EM and non-EM specialist subgroups. For the items classified as non-adopted because of persistent lack of consensus, we additionally performed a post hoc descriptive classification based on subgroup support patterns. Support was operationally defined as a subgroup median of ≥ 6, and items were classified into four patterns: neither subgroup supportive, only the EM subgroup supportive, only the non-EM subgroup supportive, or both subgroups supportive but the prespecified whole-panel adoption criteria not met. These subgroup analyses were descriptive and were not used for consensus classification, and no inferential statistical testing was performed. All analyses were conducted using IBM SPSS Statistics, version 30.0 (IBM Corp., Armonk, NY, USA).

## Results

### Panel characteristics

The Delphi panel comprised 50 specialists—25 EM and 25 non-EM—selected from 68 physicians who applied. The panel spanned all eight geographic regions of Japan, university and community hospitals, tertiary and secondary emergency facilities, and a wide range of annual emergency case volumes. Overall, 32 of 50 panellists worked in community hospitals and 31 in secondary emergency departments; among non-EM specialists, 19 of 25 worked in community hospitals and 20 of 25 in secondary emergency departments. Twenty-one panellists (42%) had previously practised in geographically isolated settings, where the nearest tertiary emergency centre was more than two hours away. Mean emergency care experience was approximately 13 years in both groups. Panel characteristics by subgroup are presented in Table 1.

**Table 1:** Panel characteristics by expertise group

Characteristic	Total (*n* = 50)	EM specialists (*n* = 25)	Non-EM specialists (*n* = 25)
Hospital type, n (%)			
University hospital	18 (36%)	12 (48%)	6 (24%)
Community hospital	32 (64%)	13 (52%)	19 (76%)
Type of emergency facility, n (%)			
Tertiary emergency centre	19 (38%)	14 (56%)	5 (20%)
Secondary emergency department	31 (62%)	11 (44%)	20 (80%)
Years of emergency care experience, mean ± SD	13.3 ± 5.3	13.0 ± 6.1	13.6 ± 4.5
Annual emergency case volume, n (%)			
≥ 1000 cases	15 (30%)	12 (48%)	3 (12%)
501–1000 cases	7 (14%)	5 (20%)	2 (8%)
301–500 cases	6 (12%)	2 (8%)	4 (16%)
201–300 cases	9 (18%)	3 (12%)	6 (24%)
< 200 cases	13 (26%)	3 (12%)	10 (40%)
Experience in settings distant from tertiary emergency care*, n (%)			
Yes	21 (42%)	10 (40%)	11 (44%)
No	29 (58%)	15 (60%)	14 (56%)
Specialty board certifications, n (multiple responses allowed)			
Emergency medicine	25	25	0
Primary care/family medicine	16	6	10
General medicine	10	2	8
Internal medicine	28	11	17
Other specialties^†^	12	8	4
Regional distribution, n			
Hokkaido	5	3	2
Tohoku	4	2	2
Kanto	10	4	6
Chubu	6	4	2
Kansai	9	5	4
Chugoku	5	1	4
Shikoku	4	3	1
Kyushu/Okinawa	7	3	4

^*^Defined as practice in settings where the nearest tertiary emergency centre was more than 2 h away

^†^Other specialties include critical care, anaesthesia, infectious disease, obstetrics & gynaecology, orthopaedics, and home care medicine

### Response rates

Of the 50 enrolled panellists, 48 completed Round 1 (96.0%), 42 of them completed Round 2 (87.5%), and 41 of those 42 completed Round 3 (97.6%); 41 of the 50 enrolled panellists (82.0%) thus completed all three rounds. The numbers of EM and non-EM specialists completing Rounds 1, 2, and 3 were 24 and 24, 22 and 20, and 21 and 20, respectively. Although attrition was not perfectly symmetrical, the two groups remained closely balanced throughout the study.

### Item flow across Delphi rounds

**Fig. 1:**
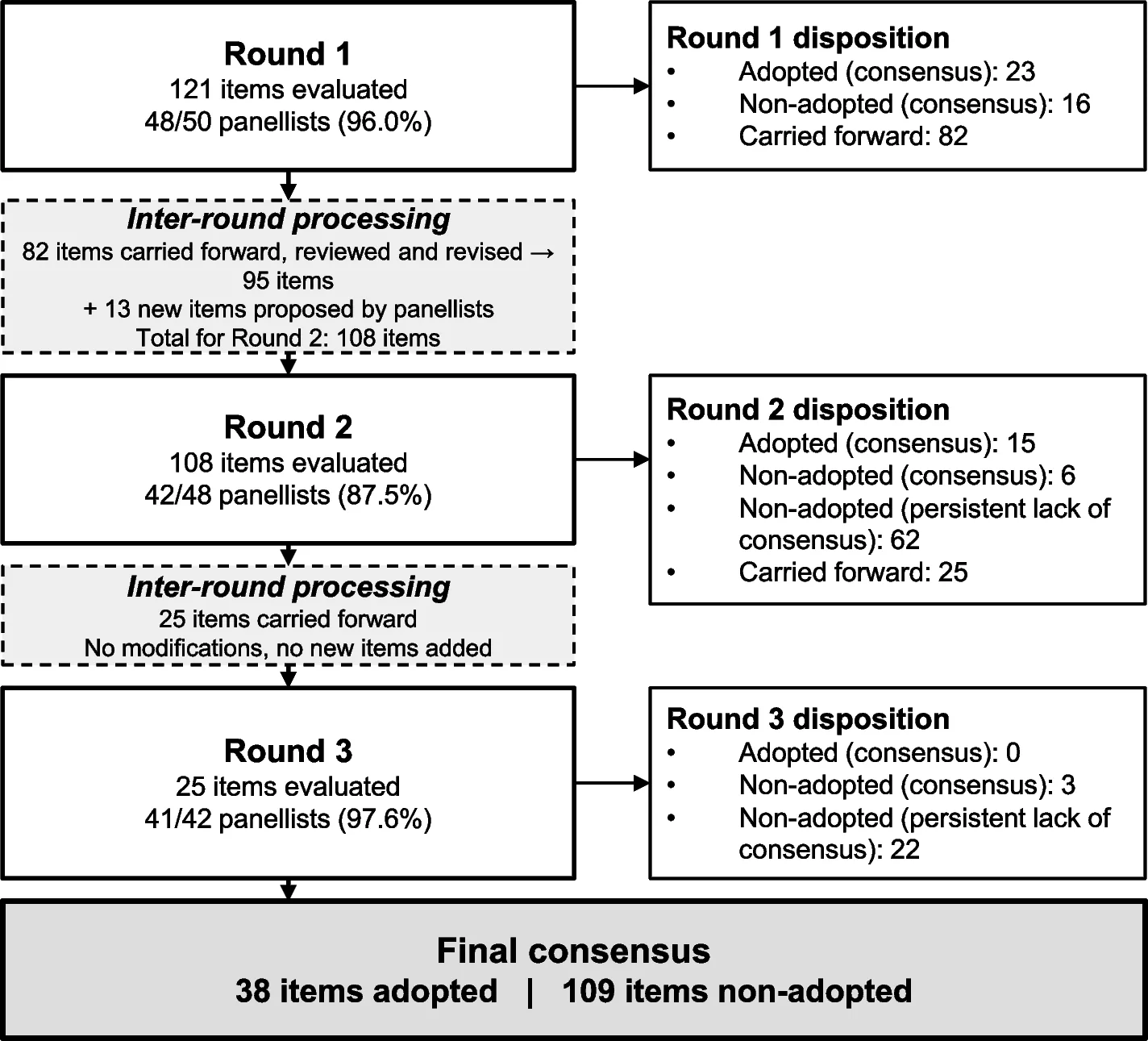
Flow of competency items through the three-round modified Delphi process. Flow of the 121 initial competency items through three Delphi rounds. For each round, the left box shows the number of items evaluated and the response rate, and the right box shows the disposition of those items. Items meeting the adoption or consensus non-adoption criteria were resolved; the remainder were carried forward. Between rounds, carried-forward items were reviewed and revised, some were split into more specific items, and panellists could propose new items. Items that twice failed to reach adoption or consensus non-adoption were classified as non-adopted by persistent lack of consensus. Across the three rounds, 38 items reached adoption consensus and 109 were non-adopted

The flow of items through the Delphi process is shown in Fig. 1. In Round 1, 121 items were evaluated: 23 met the adoption criteria and 16 met the consensus non-adoption criteria, while the remaining 82 were carried forward for inter-round revision—54 retained unchanged, 15 given minor wording revision for clarity, 3 substantively reworded, and 10 split into 23 more specific items. Panellists also proposed 13 new items during Round 1. The Round 2 pool therefore comprised 108 items: 69 undergoing a second round of rating (54 unchanged and 15 minor revisions) and 39 rated for the first time (3 reworded, 23 split-derived, and 13 newly proposed).

In Round 2, of the 108 items evaluated, 15 met the adoption criteria and 6 met the consensus non-adoption criteria. Of the 69 items rated for a second time, 62 had met neither the adoption nor the consensus non-adoption criteria across both rounds and were therefore classified as non-adopted by persistent lack of consensus. The remaining 25 items proceeded to Round 3.

In Round 3, the remaining 25 items were evaluated: none met the adoption criteria, 3 met the consensus non-adoption criteria, and the remaining 22 were classified as non-adopted by persistent lack of consensus. Item-level results for every item in each round—median, quartile deviation, and distribution of ratings—are provided in Supplementary Tables S3–S5.

### Adopted items

The 38 items that reached adoption consensus are presented by category in Table 2. At the round of adoption, the median rating was 7 for 29 of the 38 items (76.3%), the proportion of ratings in the 6–7 range exceeded 90% for 22 items (57.9%), and the quartile deviation was ≤ 0.5 for all 38 items, indicating strong panel agreement across the adopted competencies.

**Table 2:** Adopted emergency care competency items for non-emergency medicine specialists

Item	Round adopted	Median	% rated 6–7	QD
Airway				
Insert an oropharyngeal airway	R1	7.0	85.4%	0.50
Insert a nasopharyngeal airway	R1	7.0	91.7%	0.00
Perform orotracheal intubation in cardiac arrest patients using a direct laryngoscope	R2	7.0	85.7%	0.50
Perform orotracheal intubation in cardiac arrest patients using a video laryngoscope	R2	6.5	88.1%	0.50
Perform airway foreign body removal using back blows and the Heimlich manoeuvre	R2	7.0	83.3%	0.50
Perform tracheostomy tube replacement	R2	6.0	81.0%	0.50
Breathing				
Perform bag-valve-mask ventilation	R1	7.0	97.9%	0.00
Administer high-flow nasal oxygen therapy	R2	6.0	81.0%	0.50
Perform non-invasive positive pressure ventilation	R2	6.0	83.3%	0.50
Circulation				
Insert a peripheral intravenous line	R1	7.0	97.9%	0.00
Insert a central venous catheter	R1	7.0	85.4%	0.50
Manage circulation with vasopressors until handover to a specialist	R2	6.0	90.5%	0.50
Neurological assessment				
Assess consciousness using GCS/JCS	R2	7.0	97.6%	0.00
Testing procedure and interpretation				
Perform lumbar puncture	R1	7.0	85.4%	0.50
Interpret electrocardiograms	R1	7.0	85.4%	0.50
Perform arterial blood sampling	R1	7.0	100.0%	0.00
Interpret blood gas analysis	R1	7.0	97.9%	0.00
Interpret routine blood tests	R1	7.0	100.0%	0.00
Interpret cerebrospinal fluid analysis	R1	7.0	85.4%	0.50
Perform diagnostic thoracentesis for pleural effusion	R2	6.0	83.3%	0.38
Imaging interpretation				
Interpret chest radiographs	R1	7.0	91.7%	0.50
Interpret head CT scans	R1	7.0	89.6%	0.50
Interpret chest CT scans	R1	7.0	85.4%	0.50
Interpret abdominal CT scans	R1	7.0	83.3%	0.50
Perform FAST	R1	7.0	93.8%	0.50
Resuscitation procedures				
Perform basic life support (BLS)	R1	7.0	100.0%	0.00
Perform advanced cardiovascular life support (ACLS)	R1	7.0	95.8%	0.00
Indication judgement				
Assess the indication for mechanical ventilation	R2	6.5	90.5%	0.50
Assess the indication for vasopressor	R2	6.0	90.5%	0.50
Assess the indication for emergency haemodialysis	R2	6.0	90.5%	0.50
Bedside procedures				
Perform direct compression for haemorrhage control	R1	7.0	100.0%	0.00
Perform simple wound suturing	R1	7.0	89.6%	0.50
Insert a nasogastric tube	R1	7.0	93.8%	0.50
Insert an indwelling urinary catheter for urinary retention (standard technique)	R2	7.0	97.6%	0.50
Perform surface cooling for heat stroke (e.g., ice packs, evaporative cooling with mist and fans)	R2	7.0	92.9%	0.50
Perform non-invasive rewarming for hypothermia (e.g., warming blankets, forced-air warmers, warmed intravenous fluids)	R2	7.0	95.2%	0.50
Infection control				
Perform appropriate donning and doffing of personal protective equipment	R1	7.0	97.9%	0.00
Legal documentation				
Complete death certificates and postmortem examination reports	R1	7.0	97.9%	0.00

Values are reported at the round of adoption

*QD* Quartile deviation

The research team grouped the 38 adopted items post hoc into 11 categories reflecting their clinical and cognitive structure. Four categories corresponded to the first four elements of the ABCDE primary survey: airway (A; 6 items), breathing (B; 3 items), circulation (C; 3 items), and neurological assessment, corresponding to disability (D; 1 item). The remaining seven categories were testing procedure and interpretation (7 items), imaging interpretation (5 items), resuscitation procedures (2 items), indication judgement (3 items), bedside procedures (6 items), infection control (1 item), and legal documentation (1 item).

In descriptive subgroup analyses, all 38 adopted items had a median rating of ≥ 6 in both the EM and non-EM specialist subgroups (Supplementary Table S6).

### Non-adopted items

Of the 109 non-adopted items, 25 (22.9%) met the consensus non-adoption criteria and 84 (77.1%) were classified as non-adopted by persistent lack of consensus. Non-adopted items spanned multiple clinical domains, including airway management beyond the cardiac arrest setting, advanced trauma management, invasive thoracic interventions, and several specialty-specific procedures. Tracheal intubation reached adoption consensus only for cardiac arrest; intubation for other indications did not reach consensus. Among the 84 items classified as non-adopted because of persistent lack of consensus, 40 (47.6%) were supported by neither subgroup, one (1.2%) was supported only by the EM subgroup, 16 (19.0%) were supported only by the non-EM subgroup, and 27 (32.1%) were supported by both subgroups but did not meet the prespecified whole-panel adoption criteria (Table 3). Thus, persistent lack of consensus reflected heterogeneous patterns of subgroup support rather than uniformly representing disagreement between EM and non-EM specialists. Subgroup medians for all non-adopted items are provided in Supplementary Table S7.

**Table 3:** Support patterns among 84 competency items classified as non-adopted because of persistent lack of consensus

Competency item	Round	EM median	Non-EM median	Overall median	% rated 6–7
**Pattern 1. Neither group supportive—EM median < 6 and non-EM median < 6; *n* = 40 (47.6%)**					
Perform needle cricothyrotomy	R2	3.0	5.0	4.0	19.0%
Perform cricothyrotomy	R2	3.0	3.0	3.0	11.9%
Insert a chest tube	R2	5.0	5.0	5.0	38.1%
Perform bronchoscopic airway suctioning and assessment	R2	3.0	3.0	3.0	7.1%
Perform pericardiocentesis	R2	2.5	2.5	2.5	4.8%
Apply a tourniquet for haemorrhage control	R2	5.0	5.5	5.0	45.2%
Apply a pelvic binder	R2	5.0	5.0	5.0	28.6%
Reduce a shoulder dislocation	R2	5.0	4.0	5.0	23.8%
Reduce distal radius and ulna fractures	R2	4.0	3.5	4.0	2.4%
Measure intra-abdominal (bladder) pressure	R2	3.5	2.5	3.0	9.5%
Perform lung POCUS	R2	5.0	5.5	5.0	45.2%
Perform airway POCUS	R2	4.0	4.0	4.0	21.4%
Perform anoscopy	R2	5.5	5.0	5.0	45.2%
Perform gastric lavage	R2	3.5	5.0	5.0	31.0%
Insert a Sengstaken-Blakemore tube	R2	2.0	2.0	2.0	9.5%
Perform emergency haemodialysis	R2	3.0	4.0	3.5	11.9%
Insert a dialysis catheter	R2	5.0	4.5	5.0	33.3%
Perform regional anaesthesia other than digital block	R2	2.5	2.5	2.5	4.8%
Remove a corneal foreign body	R2	5.0	3.0	3.0	11.9%
Assess nystagmus using Frenzel goggles	R2	5.0	5.0	5.0	45.2%
Perform posterior nasal packing using a balloon catheter	R2	3.5	3.0	3.0	9.5%
Perform fibreoptic laryngoscopy for airway evaluation	R2	3.0	3.0	3.0	9.5%
Remove a pharyngeal or laryngeal foreign body	R2	4.5	5.0	5.0	16.7%
Perform debridement of pressure ulcers	R2	5.0	5.0	5.0	42.9%
Perform obstetric emergency care (ALSO/BLSO)	R2	4.0	4.0	4.0	19.0%
Assess the indication for hyperbaric oxygen therapy	R2	4.0	3.0	3.0	11.9%
Identify toxic substances based on toxidromes	R2	5.0	5.0	5.0	16.7%
Apply PPE for CBRN incidents	R2	4.0	4.0	4.0	14.3%
Provide online medical direction to paramedics	R2	4.0	5.0	5.0	26.2%
Assess the indication for neuromuscular blockade in non-cardiac-arrest patients	R3	5.0	5.0	5.0	26.8%
Perform orotracheal intubation in non-cardiac-arrest patients with anatomically difficult airways using a video laryngoscope	R3	3.0	5.0	4.0	22.0%
Use intubation adjunct devices (e.g., gum elastic bougie) for difficult intubation	R3	4.0	4.0	4.0	9.8%
Insert a supraglottic airway device in non-cardiac-arrest patients	R3	4.0	5.5	5.0	36.6%
Manage a pacemaker device (in collaboration with a clinical engineer)	R3	4.0	3.5	4.0	9.8%
Perform obstetric and gynaecologic POCUS	R3	3.0	3.0	3.0	9.8%
Perform urologic POCUS	R3	5.0	5.0	5.0	22.0%
Perform carotid artery POCUS	R3	4.0	5.0	5.0	22.0%
Insert an indwelling urinary catheter for urinary retention (using a guidewire)	R3	3.0	4.5	3.0	14.6%
Perform cold-water immersion (ice bath) for heat stroke	R3	4.0	5.0	4.0	31.7%
Provide initial treatment for poisoning based on the substance (supportive care and assessment of indication for specific therapy)	R3	5.0	4.5	5.0	22.0%
**Pattern 2. EM group only supportive—EM median ≥ 6 and non-EM median < 6; *n* = 1 (1.2%)**					
Assess anatomically difficult airways (e.g., using the LEMON criteria)	R3	6.0	5.5	6.0	58.5%
**Pattern 3. Non-EM group only supportive—EM median < 6 and non-EM median ≥ 6; *n* = 16 (19.0%)**					
Perform transcutaneous pacing	R2	5.0	6.0	5.0	47.6%
Insert an intraosseous needle	R2	5.5	6.0	6.0	59.5%
Insert an arterial line	R2	5.0	6.0	5.0	47.6%
Assess and reduce benign paroxysmal positional vertigo (e.g., Dix-Hallpike test, supine roll test, Epley, Gufoni, and Barbecue rotation manoeuvres)	R2	5.5	6.0	6.0	52.4%
Reduce a temporomandibular joint dislocation	R2	5.5	6.0	6.0	61.9%
Apply a backboard	R2	5.0	6.0	6.0	59.5%
Perform a foreign body removal with simple incision	R2	5.5	6.0	6.0	54.8%
Interpret spinal MRI	R2	5.5	6.0	6.0	57.1%
Perform POCUS for deep vein thrombosis	R2	5.0	6.0	5.0	47.6%
Remove a foreign body from the external auditory canal	R2	5.0	6.0	5.0	47.6%
Perform incision and drainage of an abscess	R2	5.5	6.0	6.0	57.1%
Perform Gram staining of prepared specimens	R2	5.5	6.0	6.0	57.1%
Perform paediatric advanced life support (PALS)	R2	5.0	6.0	6.0	52.4%
Perform secondary triage using the PAT system	R2	5.0	6.0	6.0	54.8%
Assess stroke severity using the NIHSS	R3	5.0	6.0	6.0	53.7%
Assess burns (depth and extent)	R3	5.0	6.0	6.0	51.2%
**Pattern 4. Both groups supportive, but whole-panel adoption criteria not met—EM median ≥ 6 and non-EM median ≥ 6; *n* = 27 (32.1%)**					
Perform synchronised cardioversion for arrhythmias	R2	6.0	6.0	6.0	54.8%
Perform vagal manoeuvres (e.g., Valsalva manoeuvre)	R2	6.0	6.0	6.0	73.8%
Reduce nursemaid's elbow	R2	6.0	6.0	6.0	76.2%
Apply a splint to the upper extremity	R2	6.0	6.0	6.0	71.4%
Apply a splint to the lower extremity	R2	6.0	6.0	6.0	71.4%
Apply a cervical collar	R2	6.0	6.0	6.0	71.4%
Interpret extremity radiographs	R2	6.0	6.0	6.0	78.6%
Interpret spinal CT scans	R2	6.0	6.0	6.0	64.3%
Interpret extremity CT scans	R2	6.0	6.0	6.0	64.3%
Interpret brain MRI	R2	6.0	6.0	6.0	78.6%
Perform cardiac POCUS	R2	6.0	6.0	6.0	66.7%
Perform abdominal POCUS	R2	6.0	6.0	6.0	73.8%
Perform paracentesis	R2	6.0	6.0	6.0	76.2%
Perform procedural sedation and analgesia	R2	6.0	6.0	6.0	78.6%
Perform a digital nerve block	R2	6.0	6.0	6.0	54.8%
Perform ocular irrigation	R2	6.0	6.0	6.0	76.2%
Perform anterior nasal packing	R2	6.0	6.0	6.0	76.2%
Perform standardised initial trauma care (JATEC)	R2	6.0	6.0	6.0	61.9%
Perform primary triage using the START system	R2	6.0	6.0	6.0	66.7%
Assess physiologically difficult airways (e.g., hypoxia, hypotension, metabolic acidosis)	R3	6.0	6.0	6.0	73.2%
Perform orotracheal intubation in non-cardiac-arrest patients without anatomical difficulty using a direct laryngoscope	R3	6.0	6.0	6.0	70.7%
Perform orotracheal intubation in non-cardiac-arrest patients without anatomical difficulty using a video laryngoscope	R3	6.0	6.0	6.0	75.6%
Insert a supraglottic airway device in cardiac arrest patients	R3	6.0	6.0	6.0	56.1%
Perform airway foreign body removal using Magill forceps under laryngoscopy	R3	6.0	6.0	6.0	63.4%
Manage mechanical ventilation until handover to a specialist	R3	6.0	6.5	6.0	75.6%
Perform needle thoracentesis for tension pneumothorax	R3	6.0	6.0	6.0	78.0%
Provide conservative management (topical agents and dressings) for mild-to-moderate burns (up to superficial partial-thickness)	R3	6.0	6.0	6.0	70.7%

For this post hoc descriptive classification, support was operationally defined as a subgroup median of ≥ 6. The prespecified adoption criteria were a whole-panel median of ≥ 6, ≥ 80% of ratings of 6–7, and a quartile deviation of ≤ 1.0. All 84 items failed to meet the adoption or consensus non-adoption criteria in two consecutive rounds

*EM* Emergency medicine specialists, *non-EM* non-emergency medicine specialists

## Discussion

### Summary of findings

In this modified Delphi study, we identified 38 core emergency care competencies for non-EM specialists who provide first-contact emergency care in Japan. The adopted competencies centred on recognising acute deterioration, interpreting laboratory and imaging findings, initial stabilisation, judging indications for specialist-level intervention, and timely escalation, together with selected bedside procedures, infection control, and legal documentation; several high-risk invasive procedures did not reach consensus as universal core competencies. All adopted items had a median rating of ≥ 6 in both the EM and non-EM specialist subgroups, indicating broad agreement across the two groups. The competencies expected of non-EM specialists in diagnostically enabled but specialist-limited settings are therefore not a reduced version of EM specialist training but a distinct, role-specific profile.

### Interpretation of findings

The adopted competencies are best understood not as a smaller version of the emergency physician's skill set but as a differently weighted one. Previous modified Delphi studies of emergency care competencies have almost all addressed physicians training to become emergency specialists and have tended towards comprehensive procedural coverage. One modified Delphi study of emergency airway management, for example, defined 148 competencies to prepare every graduating emergency resident for independent airway practice [17]. The same pattern holds in other domains: a modified Delphi for emergency ultrasound, for instance, produced a comprehensive list of competencies expected of every graduating resident [18]. Applied to non-EM specialists rather than to future emergency physicians, the same consensus method produced a selective, decision-weighted set rather than a comprehensive, procedure-weighted one.

The contrast is sharpest for tracheal intubation. Whereas competency frameworks for emergency residents treat airway management as an extensive domain to be mastered for independent practice [17], our panel adopted intubation only for cardiac arrest and reached no consensus on intubation for other indications. This boundary may reflect both the setting and the nature of the procedures. Many procedures that did not reach consensus are high-acuity, low-occurrence (HALO) skills—technically demanding but performed infrequently—for which independent proficiency is difficult to acquire and maintain without regular practice [19].

The non-adopted items nevertheless included several time-critical or clinically important competencies with direct implications for patient survival. However, the rating patterns among the 84 items classified as non-adopted because of persistent lack of consensus were not uniform. Forty items did not meet the operational support threshold in either subgroup, whereas 27 had median ratings of ≥ 6 in both the EM and non-EM subgroups but did not meet the stringent prespecified whole-panel adoption criteria. The latter group included standardised initial trauma care, procedural sedation and analgesia, orotracheal intubation in non-cardiac-arrest patients, management of mechanical ventilation until specialist handover, and needle thoracentesis for tension pneumothorax.

Accordingly, non-adoption should not be interpreted as indicating that these competencies were considered unnecessary for non-EM physicians, clinically inappropriate, or outside their scope of practice in all settings. Because panellists rated competencies as general standards rather than in relation to specific institutional or clinical contexts, the present study cannot determine the optimal course of action in an individual situation—whether a non-EM physician should perform a time-critical intervention or immediately seek specialist support.

Rather, the 38 adopted competencies should be interpreted as a minimum core intended for broad application across diverse hospital settings, rather than as an exhaustive list or an upper limit on the competencies required of non-EM physicians. The decision-weighted profile may reflect the interaction between the expected role of non-EM physicians, the clinical environments in which they practise, and the difficulty of maintaining proficiency in infrequently performed procedures. It therefore supports placing educational emphasis on recognising deterioration, judging indications for specialist-level intervention, and seeking timely specialist support and escalating care when appropriate, without implying that non-adopted competencies should be excluded from local curricula or practice settings. Institutions and regions may need to add context-specific competencies according to specialist availability, transfer time, local resources, case mix, and the frequency of time-critical emergencies. This emphasis is consistent with a wider literature that treats recognition of deterioration and escalation decisions as important determinants of acute-care outcomes in their own right [20, 21].

The subgroup analyses may offer some insight into how EM and non-EM specialists differed in their rating tendencies. Sixteen of the 84 items met the operational support threshold only in the non-EM subgroup, whereas only one met it only in the EM subgroup. The reasons for this pattern cannot be determined from the present quantitative Delphi data, but it may reflect differences in clinical exposure, perceived role relevance, or expectations regarding generalist emergency practice, and warrants further qualitative investigation.

### Educational implications

These findings have direct implications for how emergency care is taught to non-EM specialists. To our knowledge, this is the first study to define, by expert consensus, the core emergency care competencies expected of non-EM specialists in a diagnostically enabled but specialist-limited setting. Because the 38 competencies were derived through a structured consensus process with a balanced panel, they provide content validity evidence that can serve as a foundation for curriculum design, learning objectives, and assessment blueprints.

The adopted profile also indicates how such a curriculum should be weighted. A programme built on these competencies would centre not on accumulating procedural repertoire but on the cognitive core that defines the role: recognising acute deterioration, interpreting laboratory and imaging findings, judging indications for specialist-level intervention, and deciding when and how to escalate. These are not merely desirable attributes but teachable competencies: educational interventions targeting the recognition and management of deterioration improve learner, system, and patient outcomes, particularly when they incorporate simulation [22]. Escalation in particular warrants explicit instruction: although failure to escalate is a recognised contributor to adverse events, it is seldom taught as a competency in its own right, and trainees often receive no formal preparation for it [21, 23]. Education for non-EM specialists should therefore be designed not as a reduced version of emergency specialist training but as a distinct curriculum. More broadly, the same approach—defining a role by the decisions it requires, then designing education around that role—may be transferable to non-EM specialists in other high-resource systems with uneven EM specialist coverage.

The present study, however, did not establish an instructional sequence or hierarchy among the competencies. Adoption status and rating strength should therefore not be interpreted as determining the order in which competencies should be taught. Some adopted competencies, such as FAST, head CT interpretation, and central venous catheter insertion, may appear more technically advanced than some time-critical competencies that were not adopted. The Delphi survey could not determine whether this pattern reflected educational prioritisation, the composition of the panel, or the familiarity of panellists with these skills in routine practice. In Japan, ultrasonography and central venous catheter insertion are included among the objectives of postgraduate clinical training and may therefore have been regarded by panellists as established components of general clinical practice rather than as advanced specialist skills. [24]

When translating the framework into a curriculum, foundational assessment, initial stabilisation, and time-critical life-saving skills should be taught before or alongside diagnostic and procedural competencies. The sequence and depth of instruction should be determined by learner experience, local case mix, available supervision, specialist accessibility, and institutional resources rather than by the order or magnitude of Delphi ratings alone. Further qualitative research exploring the reasoning underlying panellists’ ratings may help clarify how these competencies should be prioritised and sequenced across different clinical settings.

A curriculum is defined not only by its content but by when it is delivered. In Japan, non-EM specialists provide first-contact emergency care before or alongside their formal specialty training, so this education is best introduced early—during supervised pre-specialty training—and sustained as continuing professional development. Cultivating foundational competencies before or during specialty differentiation is not unique to emergency care: geriatric medicine has responded to specialist shortage with a parallel effort to build core competencies in all physicians, introduced early in training [25].

### Limitations

This study has several limitations. First, it defined a consensus-based set of competencies but did not implement or evaluate them; the 38 items represent content validity evidence for what should be taught, not evidence that teaching them improves performance or patient outcomes. Their translation into curricula and assessments, and their educational effects, remain to be tested. Second, the study was conducted within a single country and health system, and the competencies reflect the Japanese context; their transferability to other systems is therefore a hypothesis rather than an established finding. Third, the 11-category structure used to characterise the adopted items was applied post hoc by the research team and did not form part of the Delphi consensus process; it is an organising aid rather than a consensus output. Fourth, the results depend on panel composition and selection criteria, including the annual case-volume threshold set by the research team; a differently constituted panel might have produced a different set of competencies. We also did not collect data on panellists’ age or sex/gender and therefore could not assess the sociodemographic diversity of the panel. Fifth, the subgroup support-pattern analysis was post hoc and descriptive, without inferential statistical testing, and should therefore be interpreted as exploratory. Finally, because competencies were rated as general standards rather than within specific institutional or clinical scenarios, the study cannot determine context-specific scope of practice, the optimal balance between performing an intervention and seeking specialist support, or the appropriate instructional sequence.

## Conclusions

Using a modified Delphi process with a balanced panel of EM and non-EM specialists, we defined 38 core emergency care competencies for non-EM specialists who provide first-contact emergency care in diagnostically enabled but specialist-limited settings. The competencies that reached consensus were weighted towards recognition, interpretation, judgement, and escalation rather than comprehensive procedural coverage, indicating that the role is not a reduced version of emergency specialist practice but a distinct, decision-centred one. These competencies provide a content-valid minimum core for designing education aligned with the role non-EM specialists are expected to perform.

## Supplementary Information

Supplementary Material 1

Supplementary Material 2

Supplementary Material 3

Supplementary Material 4

Supplementary Material 5

Supplementary Material 6

Supplementary Material 7

## Data Availability

Aggregated datasets supporting the findings of this study are included in the supplementary information files (Supplementary Tables S2–S7). The individual-level response data are not publicly available because they could compromise participant confidentiality, but may be available from the corresponding author on reasonable request, subject to ethical approval and applicable data protection requirements.

## Abbreviations

ABCDE: Airway, breathing, circulation, disability, exposure
CREDES: Conducting and REporting of DElphi Studies
EM: Emergency medicine
HALO: High-acuity, low-occurrence
JAAM: Japanese Association for Acute Medicine
QD: Quartile deviation
