# Supplementary material for: Emergency care competencies for non–emergency medicine specialists in Japan: a modified Delphi study
Source: BMC Med Educ. 2026 Aug 8;26:1786. doi: 10.1186/s12909-026-10094-0 (PMC13732881; doi:10.1186/s12909-026-10094-0)
Supplement: Supplementary file 2 [file 12909_2026_10094_MOESM2_ESM.docx]

# Supplementary Table S2

# Round 1 Delphi Questionnaire

## Instructions to respondents

For each item listed below, please rate the extent to which you agree that this is a skill that non-EM specialists should acquire, to a level at which they could perform it independently — that is, without relying on another medical specialist, while assuming routine clinical support such as nursing assistance.

Please do not assume any specific institutional level. Rate each item as a normative general standard rather than according to current practice at your own institution.

## Response scale

Respondents rated each item on a 7-point Likert scale:

1 — Strongly disagree

2 — Disagree

3 — Slightly disagree

4 — Neither agree nor disagree

5 — Slightly agree

6 — Agree

7 — Strongly agree

## Round 1 items (n = 121)

| **No.** | **Item** |
| --- | --- |
| 1 | Perform standard orotracheal intubation |
| 2 | Perform orotracheal intubation in anatomically difficult airways |
| 3 | Perform rapid sequence intubation |
| 4 | Insert an oropharyngeal airway |
| 5 | Insert a nasopharyngeal airway |
| 6 | Insert a supraglottic airway device |
| 7 | Perform fibreoptic nasotracheal intubation |
| 8 | Perform needle cricothyrotomy |
| 9 | Perform cricothyrotomy |
| 10 | Perform tracheostomy |
| 11 | Remove an airway foreign body |
| 12 | Perform bag-valve-mask ventilation |
| 13 | Administer high-flow nasal oxygen therapy |
| 14 | Perform non-invasive positive pressure ventilation |
| 15 | Initiate and manage mechanical ventilation |
| 16 | Perform thoracentesis |
| 17 | Insert a chest tube |
| 18 | Perform thoracotomy |
| 19 | Perform bronchoscopic airway suctioning and assessment |
| 20 | Perform open-chest cardiac massage |
| 21 | Perform synchronised cardioversion for arrhythmias |
| 22 | Perform transcutaneous pacing |
| 23 | Perform transvenous pacing |
| 24 | Manage a pacemaker device |
| 25 | Perform pericardiocentesis |
| 26 | Perform pericardial window |
| 27 | Insert a peripheral intravenous line |
| 28 | Insert a central venous catheter |
| 29 | Insert an intraosseous needle |
| 30 | Insert an arterial line |
| 31 | Insert a pulmonary artery catheter |
| 32 | Initiate and manage intra-aortic balloon pumping |
| 33 | Initiate and manage extracorporeal membrane oxygenation |
| 34 | Insert a REBOA (resuscitative endovascular balloon occlusion of the aorta) catheter |
| 35 | Perform vagal manoeuvres (e.g., Valsalva manoeuvre) |
| 36 | Perform lumbar puncture |
| 37 | Perform the Epley manoeuvre for benign paroxysmal positional vertigo |
| 38 | Insert an intracranial pressure monitor |
| 39 | Perform direct compression for haemorrhage control |
| 40 | Apply a tourniquet for haemorrhage control |
| 41 | Apply a pelvic binder |
| 42 | Reduce a shoulder dislocation |
| 43 | Reduce a hip dislocation |
| 44 | Reduce nursemaid's elbow |
| 45 | Reduce a temporomandibular joint dislocation |
| 46 | Reduce distal radius and ulna fractures |
| 47 | Apply a splint to the upper extremity |
| 48 | Apply a splint to the lower extremity |
| 49 | Apply traction to the lower extremity |
| 50 | Apply a cervical collar |
| 51 | Apply a backboard |
| 52 | Perform simple wound suturing |
| 53 | Perform a foreign body removal with simple incision |
| 54 | Manage burn wounds |
| 55 | Perform escharotomy |
| 56 | Interpret electrocardiograms |
| 57 | Perform arterial blood sampling |
| 58 | Interpret blood gas analysis |
| 59 | Interpret routine blood tests |
| 60 | Interpret cerebrospinal fluid analysis |
| 61 | Measure intra-abdominal (bladder) pressure |
| 62 | Measure intracompartmental pressure |
| 63 | Interpret chest radiographs |
| 64 | Interpret extremity radiographs |
| 65 | Interpret head CT scans |
| 66 | Interpret chest CT scans |
| 67 | Interpret abdominal CT scans |
| 68 | Interpret spinal CT scans |
| 69 | Interpret extremity CT scans |
| 70 | Interpret brain MRI |
| 71 | Interpret spinal MRI |
| 72 | Perform FAST (Focused Assessment with Sonography for Trauma) |
| 73 | Perform cardiac POCUS |
| 74 | Perform lung POCUS |
| 75 | Perform airway POCUS |
| 76 | Perform abdominal POCUS |
| 77 | Perform POCUS for deep vein thrombosis |
| 78 | Perform anoscopy |
| 79 | Insert a nasogastric tube |
| 80 | Perform gastric lavage |
| 81 | Insert an intestinal decompression tube |
| 82 | Insert a Sengstaken–Blakemore tube |
| 83 | Perform paracentesis |
| 84 | Assess the indication for emergency haemodialysis |
| 85 | Perform emergency haemodialysis |
| 86 | Insert a dialysis catheter |
| 87 | Perform suprapubic bladder puncture |
| 88 | Assist vaginal delivery |
| 89 | Perform procedural sedation and analgesia |
| 90 | Perform a digital nerve block |
| 91 | Perform regional anaesthesia other than digital block |
| 92 | Perform ocular irrigation |
| 93 | Perform slit-lamp examination of the cornea |
| 94 | Remove a corneal foreign body |
| 95 | Perform lateral canthotomy |
| 96 | Assess nystagmus using Frenzel goggles |
| 97 | Remove a foreign body from the external auditory canal |
| 98 | Perform anterior nasal packing |
| 99 | Perform posterior nasal packing using a balloon catheter |
| 100 | Perform fibreoptic laryngoscopy for airway evaluation |
| 101 | Remove a pharyngeal or laryngeal foreign body |
| 102 | Perform incision and drainage of an abscess |
| 103 | Perform debridement of pressure ulcers |
| 104 | Perform appropriate donning and doffing of personal protective equipment |
| 105 | Perform Gram staining of prepared specimens |
| 106 | Perform basic life support (BLS) |
| 107 | Perform advanced cardiovascular life support (ACLS) |
| 108 | Perform paediatric advanced life support (PALS) |
| 109 | Perform standardised initial trauma care (JATEC) |
| 110 | Perform obstetric emergency care (ALSO/BLSO) |
| 111 | Perform active cooling for heat stroke |
| 112 | Perform rewarming for hypothermia |
| 113 | Assess the indication for hyperbaric oxygen therapy |
| 114 | Identify toxic substances based on toxidromes |
| 115 | Perform initial treatment for poisoning |
| 116 | Apply PPE for CBRN incidents |
| 117 | Perform primary triage using the START system |
| 118 | Perform secondary triage using the PAT system |
| 119 | Complete death certificates and postmortem examination reports |
| 120 | Perform and assess neurological examinations for brain death determination |
| 121 | Provide online medical direction to paramedics |
