# Supplementary material for: Emergency care competencies for non–emergency medicine specialists in Japan: a modified Delphi study
Source: BMC Med Educ. 2026 Aug 8;26:1786. doi: 10.1186/s12909-026-10094-0 (PMC13732881; doi:10.1186/s12909-026-10094-0)
Supplement: Supplementary file 3 [file 12909_2026_10094_MOESM3_ESM.docx]

**Supplemental Table S3. Round 1 results**

| **Item** | **Round count** | **Median** | **% rated 6–7** | **% rated 1–3** | **QD** | **Consensus category** | **Next round** |
| --- | --- | --- | --- | --- | --- | --- | --- |
| Perform standard orotracheal intubation | 1st time | 7 | 79.2% | 4.2% | 0.50 | Pending (carried to Round 2) | Split |
| Perform orotracheal intubation in anatomically difficult airways | 1st time | 4 | 12.5% | 41.7% | 1.00 | Pending (carried to Round 2) | Split |
| Perform rapid sequence intubation | 1st time | 5 | 27.1% | 27.1% | 1.50 | Pending (carried to Round 2) | Split |
| Insert an oropharyngeal airway | 1st time | 7 | 85.4% | 2.1% | 0.50 | Adopted | — |
| Insert a nasopharyngeal airway | 1st time | 7 | 91.7% | 0.0% | 0.00 | Adopted | — |
| Insert a supraglottic airway device | 1st time | 6 | 54.2% | 10.4% | 1.00 | Pending (carried to Round 2) | Split |
| Perform fibreoptic nasotracheal intubation | 1st time | 2 | 6.3% | 72.9% | 1.50 | Non-adopted (consensus) | — |
| Perform needle cricothyrotomy | 1st time | 4 | 18.8% | 45.8% | 2.00 | Pending (carried to Round 2) | Continue |
| Perform cricothyrotomy | 1st time | 3 | 6.3% | 58.3% | 1.25 | Pending (carried to Round 2) | Continue |
| Perform tracheostomy | 1st time | 1 | 0.0% | 91.7% | 0.50 | Non-adopted (consensus) | — |
| Remove an airway foreign body | 1st time | 5 | 43.8% | 22.9% | 1.00 | Pending (carried to Round 2) | Split |
| Perform bag-valve-mask ventilation | 1st time | 7 | 97.9% | 0.0% | 0.00 | Adopted | — |
| Administer high-flow nasal oxygen therapy | 1st time | 6 | 72.9% | 8.3% | 1.00 | Pending (carried to Round 2) | Continue |
| Perform non-invasive positive pressure ventilation | 1st time | 6 | 64.6% | 12.5% | 1.00 | Pending (carried to Round 2) | Continue |
| Initiate and manage mechanical ventilation | 1st time | 6 | 62.5% | 12.5% | 1.00 | Pending (carried to Round 2) | Split |
| Perform thoracentesis | 1st time | 6 | 72.9% | 8.3% | 1.00 | Pending (carried to Round 2) | Split |
| Insert a chest tube | 1st time | 5 | 41.7% | 18.8% | 1.00 | Pending (carried to Round 2) | Modify |
| Perform thoracotomy | 1st time | 1 | 0.0% | 97.9% | 0.00 | Non-adopted (consensus) | — |
| Perform bronchoscopic airway suctioning and assessment | 1st time | 3 | 4.2% | 58.3% | 1.50 | Pending (carried to Round 2) | Continue |
| Perform open-chest cardiac massage | 1st time | 1 | 0.0% | 100.0% | 0.00 | Non-adopted (consensus) | — |
| Perform synchronised cardioversion for arrhythmias | 1st time | 6 | 52.1% | 12.5% | 0.50 | Pending (carried to Round 2) | Continue |
| Perform transcutaneous pacing | 1st time | 5 | 43.8% | 14.6% | 0.50 | Pending (carried to Round 2) | Continue |
| Perform transvenous pacing | 1st time | 1 | 0.0% | 95.8% | 0.50 | Non-adopted (consensus) | — |
| Manage a pacemaker device | 1st time | 2.5 | 2.1% | 62.5% | 1.50 | Pending (carried to Round 2) | Modify |
| Perform pericardiocentesis | 1st time | 2 | 4.2% | 68.8% | 1.50 | Pending (carried to Round 2) | Continue |
| Perform pericardial window | 1st time | 1 | 0.0% | 100.0% | 0.50 | Non-adopted (consensus) | — |
| Insert a peripheral intravenous line | 1st time | 7 | 97.9% | 2.1% | 0.00 | Adopted | — |
| Insert a central venous catheter | 1st time | 7 | 85.4% | 4.2% | 0.50 | Adopted | — |
| Insert an intraosseous needle | 1st time | 6 | 68.8% | 6.3% | 1.00 | Pending (carried to Round 2) | Continue |
| Insert an arterial line | 1st time | 5 | 47.9% | 20.8% | 1.00 | Pending (carried to Round 2) | Continue |
| Insert a pulmonary artery catheter | 1st time | 1 | 0.0% | 89.6% | 1.00 | Non-adopted (consensus) | — |
| Initiate and manage intra-aortic balloon pumping | 1st time | 1 | 0.0% | 95.8% | 0.50 | Non-adopted (consensus) | — |
| Initiate and manage extracorporeal membrane oxygenation | 1st time | 1 | 0.0% | 97.9% | 0.13 | Non-adopted (consensus) | — |
| Insert a REBOA (resuscitative endovascular balloon occlusion of the aorta) catheter | 1st time | 1 | 0.0% | 91.7% | 0.50 | Non-adopted (consensus) | — |
| Perform vagal manoeuvres (e.g., Valsalva manoeuvre) | 1st time | 6.5 | 70.8% | 0.0% | 1.00 | Pending (carried to Round 2) | Continue |
| Perform lumbar puncture | 1st time | 7 | 85.4% | 6.3% | 0.50 | Adopted | — |
| Perform the Epley manoeuvre for benign paroxysmal positional vertigo | 1st time | 6 | 52.1% | 10.4% | 0.75 | Pending (carried to Round 2) | Modify |
| Insert an intracranial pressure monitor | 1st time | 1 | 0.0% | 93.8% | 0.50 | Non-adopted (consensus) | — |
| Perform direct compression for haemorrhage control | 1st time | 7 | 100.0% | 0.0% | 0.00 | Adopted | — |
| Apply a tourniquet for haemorrhage control | 1st time | 6 | 52.1% | 14.6% | 0.50 | Pending (carried to Round 2) | Continue |
| Apply a pelvic binder | 1st time | 5 | 27.1% | 18.8% | 1.00 | Pending (carried to Round 2) | Continue |
| Reduce a shoulder dislocation | 1st time | 4 | 22.9% | 27.1% | 1.00 | Pending (carried to Round 2) | Continue |
| Reduce a hip dislocation | 1st time | 3 | 4.2% | 72.9% | 1.00 | Non-adopted (consensus) | — |
| Reduce nursemaid's elbow | 1st time | 6 | 72.9% | 4.2% | 1.00 | Pending (carried to Round 2) | Continue |
| Reduce a temporomandibular joint dislocation | 1st time | 6 | 58.3% | 6.3% | 0.50 | Pending (carried to Round 2) | Continue |
| Reduce distal radius and ulna fractures | 1st time | 4 | 8.3% | 47.9% | 1.50 | Pending (carried to Round 2) | Continue |
| Apply a splint to the upper extremity | 1st time | 6 | 72.9% | 2.1% | 1.00 | Pending (carried to Round 2) | Continue |
| Apply a splint to the lower extremity | 1st time | 6 | 72.9% | 2.1% | 1.00 | Pending (carried to Round 2) | Continue |
| Apply traction to the lower extremity | 1st time | 3 | 6.3% | 62.5% | 1.00 | Pending (carried to Round 2) | Continue |
| Apply a cervical collar | 1st time | 6.5 | 70.8% | 4.2% | 1.00 | Pending (carried to Round 2) | Continue |
| Apply a backboard | 1st time | 6 | 70.8% | 6.3% | 1.00 | Pending (carried to Round 2) | Continue |
| Perform simple wound suturing | 1st time | 7 | 89.6% | 0.0% | 0.50 | Adopted | — |
| Perform a foreign body removal with simple incision | 1st time | 6 | 62.5% | 6.3% | 0.63 | Pending (carried to Round 2) | Continue |
| Manage burn wounds | 1st time | 5 | 47.9% | 8.3% | 0.50 | Pending (carried to Round 2) | Split |
| Perform escharotomy | 1st time | 2 | 0.0% | 81.3% | 1.00 | Non-adopted (consensus) | — |
| Interpret electrocardiograms | 1st time | 7 | 85.4% | 0.0% | 0.50 | Adopted | — |
| Perform arterial blood sampling | 1st time | 7 | 100.0% | 0.0% | 0.00 | Adopted | — |
| Interpret blood gas analysis | 1st time | 7 | 97.9% | 0.0% | 0.00 | Adopted | — |
| Interpret routine blood tests | 1st time | 7 | 100.0% | 0.0% | 0.00 | Adopted | — |
| Interpret cerebrospinal fluid analysis | 1st time | 7 | 85.4% | 0.0% | 0.50 | Adopted | — |
| Measure intra-abdominal (bladder) pressure | 1st time | 4 | 10.4% | 43.8% | 1.50 | Pending (carried to Round 2) | Continue |
| Measure intracompartmental pressure | 1st time | 3 | 6.3% | 56.3% | 1.00 | Pending (carried to Round 2) | Continue |
| Interpret chest radiographs | 1st time | 7 | 91.7% | 0.0% | 0.50 | Adopted | — |
| Interpret extremity radiographs | 1st time | 6 | 75.0% | 2.1% | 0.63 | Pending (carried to Round 2) | Continue |
| Interpret head CT scans | 1st time | 7 | 89.6% | 0.0% | 0.50 | Adopted | — |
| Interpret chest CT scans | 1st time | 7 | 85.4% | 0.0% | 0.50 | Adopted | — |
| Interpret abdominal CT scans | 1st time | 7 | 83.3% | 0.0% | 0.50 | Adopted | — |
| Interpret spinal CT scans | 1st time | 5.5 | 50.0% | 8.3% | 1.00 | Pending (carried to Round 2) | Modify |
| Interpret extremity CT scans | 1st time | 5 | 47.9% | 12.5% | 1.13 | Pending (carried to Round 2) | Modify |
| Interpret brain MRI | 1st time | 6 | 75.0% | 2.1% | 0.63 | Pending (carried to Round 2) | Modify |
| Interpret spinal MRI | 1st time | 5 | 41.7% | 14.6% | 1.00 | Pending (carried to Round 2) | Modify |
| Perform FAST (Focused Assessment with Sonography for Trauma) | 1st time | 7 | 93.8% | 0.0% | 0.50 | Adopted | — |
| Perform cardiac POCUS | 1st time | 6 | 70.8% | 4.2% | 1.00 | Pending (carried to Round 2) | Modify |
| Perform lung POCUS | 1st time | 5.5 | 50.0% | 8.3% | 1.00 | Pending (carried to Round 2) | Modify |
| Perform airway POCUS | 1st time | 5 | 29.2% | 29.2% | 1.50 | Pending (carried to Round 2) | Modify |
| Perform abdominal POCUS | 1st time | 6 | 62.5% | 6.3% | 1.00 | Pending (carried to Round 2) | Modify |
| Perform POCUS for deep vein thrombosis | 1st time | 6 | 52.1% | 12.5% | 0.50 | Pending (carried to Round 2) | Modify |
| Perform anoscopy | 1st time | 5 | 45.8% | 16.7% | 1.13 | Pending (carried to Round 2) | Continue |
| Insert a nasogastric tube | 1st time | 7 | 93.8% | 2.1% | 0.50 | Adopted | — |
| Perform gastric lavage | 1st time | 5 | 37.5% | 22.9% | 1.00 | Pending (carried to Round 2) | Continue |
| Insert an intestinal decompression tube | 1st time | 3 | 6.3% | 56.3% | 1.00 | Pending (carried to Round 2) | Modify |
| Insert a Sengstaken–Blakemore tube | 1st time | 3 | 8.3% | 64.6% | 1.25 | Pending (carried to Round 2) | Continue |
| Perform paracentesis | 1st time | 6 | 66.7% | 8.3% | 1.00 | Pending (carried to Round 2) | Continue |
| Assess the indication for emergency haemodialysis | 1st time | 6 | 79.2% | 2.1% | 0.50 | Pending (carried to Round 2) | Continue |
| Perform emergency haemodialysis | 1st time | 3 | 12.5% | 52.1% | 2.00 | Pending (carried to Round 2) | Continue |
| Insert a dialysis catheter | 1st time | 5 | 35.4% | 35.4% | 1.50 | Pending (carried to Round 2) | Continue |
| Perform suprapubic bladder puncture | 1st time | 3 | 4.2% | 75.0% | 0.63 | Non-adopted (consensus) | — |
| Assist vaginal delivery | 1st time | 2 | 2.1% | 70.8% | 1.00 | Non-adopted (consensus) | — |
| Perform procedural sedation and analgesia | 1st time | 6 | 72.9% | 2.1% | 1.00 | Pending (carried to Round 2) | Continue |
| Perform a digital nerve block | 1st time | 5 | 45.8% | 8.3% | 0.50 | Pending (carried to Round 2) | Continue |
| Perform regional anaesthesia other than digital block | 1st time | 3 | 6.3% | 60.4% | 1.13 | Pending (carried to Round 2) | Continue |
| Perform ocular irrigation | 1st time | 5.5 | 50.0% | 16.7% | 0.63 | Pending (carried to Round 2) | Continue |
| Perform slit-lamp examination of the cornea | 1st time | 3 | 6.3% | 60.4% | 1.00 | Pending (carried to Round 2) | Continue |
| Remove a corneal foreign body | 1st time | 3 | 14.6% | 52.1% | 1.50 | Pending (carried to Round 2) | Continue |
| Perform lateral canthotomy | 1st time | 1 | 2.1% | 93.8% | 0.50 | Non-adopted (consensus) | — |
| Assess nystagmus using Frenzel goggles | 1st time | 5 | 43.8% | 14.6% | 1.00 | Pending (carried to Round 2) | Continue |
| Remove a foreign body from the external auditory canal | 1st time | 5 | 45.8% | 8.3% | 0.50 | Pending (carried to Round 2) | Continue |
| Perform anterior nasal packing | 1st time | 6 | 66.7% | 0.0% | 0.63 | Pending (carried to Round 2) | Continue |
| Perform posterior nasal packing using a balloon catheter | 1st time | 3 | 6.3% | 54.2% | 1.13 | Pending (carried to Round 2) | Continue |
| Perform fibreoptic laryngoscopy for airway evaluation | 1st time | 3.5 | 6.3% | 50.0% | 1.50 | Pending (carried to Round 2) | Continue |
| Remove a pharyngeal or laryngeal foreign body | 1st time | 4 | 16.7% | 41.7% | 1.00 | Pending (carried to Round 2) | Continue |
| Perform incision and drainage of an abscess | 1st time | 5 | 47.9% | 10.4% | 0.50 | Pending (carried to Round 2) | Continue |
| Perform debridement of pressure ulcers | 1st time | 5 | 35.4% | 29.2% | 1.50 | Pending (carried to Round 2) | Continue |
| Perform appropriate donning and doffing of personal protective equipment | 1st time | 7 | 97.9% | 0.0% | 0.00 | Adopted | — |
| Perform Gram staining of prepared specimens | 1st time | 5.5 | 50.0% | 10.4% | 1.13 | Pending (carried to Round 2) | Modify |
| Perform basic life support (BLS) | 1st time | 7 | 100.0% | 0.0% | 0.00 | Adopted | — |
| Perform advanced cardiovascular life support (ACLS) | 1st time | 7 | 95.8% | 0.0% | 0.00 | Adopted | — |
| Perform paediatric advanced life support (PALS) | 1st time | 6 | 52.1% | 12.5% | 0.50 | Pending (carried to Round 2) | Modify |
| Perform standardised initial trauma care (JATEC) | 1st time | 6 | 58.3% | 12.5% | 0.50 | Pending (carried to Round 2) | Modify |
| Perform obstetric emergency care (ALSO/BLSO) | 1st time | 4 | 16.7% | 25.0% | 0.63 | Pending (carried to Round 2) | Modify |
| Perform active cooling for heat stroke | 1st time | 7 | 79.2% | 0.0% | 0.50 | Pending (carried to Round 2) | Split |
| Perform rewarming for hypothermia | 1st time | 6 | 68.8% | 2.1% | 1.00 | Pending (carried to Round 2) | Split |
| Assess the indication for hyperbaric oxygen therapy | 1st time | 4.5 | 20.8% | 39.6% | 1.50 | Pending (carried to Round 2) | Continue |
| Identify toxic substances based on toxidromes | 1st time | 5 | 25.0% | 22.9% | 0.63 | Pending (carried to Round 2) | Continue |
| Perform initial treatment for poisoning | 1st time | 5 | 33.3% | 20.8% | 1.00 | Pending (carried to Round 2) | Modify |
| Apply PPE for CBRN incidents | 1st time | 4 | 22.9% | 35.4% | 1.00 | Pending (carried to Round 2) | Continue |
| Perform primary triage using the START system | 1st time | 6 | 64.6% | 12.5% | 1.00 | Pending (carried to Round 2) | Continue |
| Perform secondary triage using the PAT system | 1st time | 6 | 52.1% | 18.8% | 0.63 | Pending (carried to Round 2) | Continue |
| Complete death certificates and postmortem examination reports | 1st time | 7 | 97.9% | 0.0% | 0.00 | Adopted | — |
| Perform and assess neurological examinations for brain death determination | 1st time | 2.5 | 14.6% | 62.5% | 1.63 | Pending (carried to Round 2) | Continue |
| Provide online medical direction to paramedics | 1st time | 5 | 29.2% | 22.9% | 1.00 | Pending (carried to Round 2) | Continue |

**Legend and summary**

| **Round count** | |
| --- | --- |
| **1st time** | All Round 1 items were evaluated for the first time. |
| **Consensus category** | |
| **Adopted** | ≥80% scoring 6–7, median ≥6, quartile deviation ≤1 |
| **Non-adopted (consensus)** | ≥70% scoring 1–3, median ≤3, quartile deviation ≤2 |
| **Pending (carried to Round 2)** | Neither adoption nor consensus non-adoption criteria met in this round; carried over to Round 2. |
| **Next round** | |
| **Continue** | Carried to Round 2 without wording change. |
| **Modify** | Carried to Round 2 with minor wording revision. |
| **Split** | Divided into more specific items for Round 2. |
| **—** | Resolved in Round 1 (adopted or consensus non-adoption). |
| **Round 1 summary** | |
| **Total items evaluated** | 121 |
| **Adopted** | 23 |
| **Non-adopted (consensus)** | 16 |
| **Carried forward** | 82 (Continue 54 + Modify 18 + Split 10) |
| **Response rate** | 48 / 50 (96.0%) |
