# Supplementary material for: Emergency care competencies for non–emergency medicine specialists in Japan: a modified Delphi study
Source: BMC Med Educ. 2026 Aug 8;26:1786. doi: 10.1186/s12909-026-10094-0 (PMC13732881; doi:10.1186/s12909-026-10094-0)
Supplement: Supplementary file 4 [file 12909_2026_10094_MOESM4_ESM.docx]

**Supplemental Table S4. Round 2 results**

| **Item** | **Round count** | **Median** | **% rated 6–7** | **% rated 1–3** | **QD** | **Consensus category** | **Next round** |
| --- | --- | --- | --- | --- | --- | --- | --- |
| Assess anatomically difficult airways (e.g., using the LEMON criteria) | 1st time | 5.5 | 50.0% | 9.5% | 0.50 | Pending (carried to Round 3) | Carried to Round 3 |
| Assess physiologically difficult airways (e.g., hypoxia, hypotension, metabolic acidosis) | 1st time | 6 | 66.7% | 2.4% | 1.00 | Pending (carried to Round 3) | Carried to Round 3 |
| Assess the indication for neuromuscular blockade in non-cardiac-arrest patients | 1st time | 5 | 26.2% | 23.8% | 0.88 | Pending (carried to Round 3) | Carried to Round 3 |
| Perform orotracheal intubation in cardiac arrest patients using a direct laryngoscope | 1st time | 7 | 85.7% | 2.4% | 0.50 | Adopted | — |
| Perform orotracheal intubation in cardiac arrest patients using a video laryngoscope | 1st time | 6.5 | 88.1% | 2.4% | 0.50 | Adopted | — |
| Perform orotracheal intubation in non-cardiac-arrest patients without anatomical difficulty using a direct laryngoscope | 1st time | 6 | 66.7% | 11.9% | 1.00 | Pending (carried to Round 3) | Carried to Round 3 |
| Perform orotracheal intubation in non-cardiac-arrest patients without anatomical difficulty using a video laryngoscope | 1st time | 6 | 69.0% | 7.1% | 1.00 | Pending (carried to Round 3) | Carried to Round 3 |
| Perform orotracheal intubation in non-cardiac-arrest patients with anatomically difficult airways using a video laryngoscope | 1st time | 4 | 28.6% | 35.7% | 1.50 | Pending (carried to Round 3) | Carried to Round 3 |
| Use intubation adjunct devices (e.g., gum elastic bougie) for difficult intubation | 1st time | 4 | 14.3% | 45.2% | 1.00 | Pending (carried to Round 3) | Carried to Round 3 |
| Insert a supraglottic airway device in cardiac arrest patients | 1st time | 6 | 61.9% | 14.3% | 1.25 | Pending (carried to Round 3) | Carried to Round 3 |
| Insert a supraglottic airway device in non-cardiac-arrest patients | 1st time | 5 | 38.1% | 28.6% | 1.50 | Pending (carried to Round 3) | Carried to Round 3 |
| Perform needle cricothyrotomy | 2nd time | 4 | 19.0% | 45.2% | 1.38 | Non-adopted (persistent lack of consensus) | — |
| Perform cricothyrotomy | 2nd time | 3 | 11.9% | 64.3% | 1.00 | Non-adopted (persistent lack of consensus) | — |
| Perform airway foreign body removal using back blows and the Heimlich manoeuvre | 1st time | 7 | 83.3% | 0.0% | 0.50 | Adopted | — |
| Perform airway foreign body removal using Magill forceps under laryngoscopy | 1st time | 6 | 52.4% | 0.0% | 0.50 | Pending (carried to Round 3) | Carried to Round 3 |
| Perform tracheostomy tube replacement | 1st time | 6 | 81.0% | 0.0% | 0.50 | Adopted | — |
| Administer high-flow nasal oxygen therapy | 2nd time | 6 | 81.0% | 7.1% | 0.50 | Adopted | — |
| Perform non-invasive positive pressure ventilation | 2nd time | 6 | 83.3% | 4.8% | 0.50 | Adopted | — |
| Assess the indication for mechanical ventilation | 1st time | 6.5 | 90.5% | 2.4% | 0.50 | Adopted | — |
| Manage mechanical ventilation until handover to a specialist | 1st time | 6 | 78.6% | 7.1% | 0.50 | Pending (carried to Round 3) | Carried to Round 3 |
| Perform needle thoracentesis for tension pneumothorax | 1st time | 6 | 64.3% | 4.8% | 1.00 | Pending (carried to Round 3) | Carried to Round 3 |
| Perform diagnostic thoracentesis for pleural effusion | 1st time | 6 | 83.3% | 4.8% | 0.38 | Adopted | — |
| Insert a chest tube | 2nd time | 5 | 38.1% | 19.0% | 0.88 | Non-adopted (persistent lack of consensus) | — |
| Perform bronchoscopic airway suctioning and assessment | 2nd time | 3 | 7.1% | 57.1% | 1.50 | Non-adopted (persistent lack of consensus) | — |
| Perform synchronised cardioversion for arrhythmias | 2nd time | 6 | 54.8% | 7.1% | 0.50 | Non-adopted (persistent lack of consensus) | — |
| Perform transcutaneous pacing | 2nd time | 5 | 47.6% | 11.9% | 0.50 | Non-adopted (persistent lack of consensus) | — |
| Manage a pacemaker device (in collaboration with a clinical engineer) | 1st time | 4 | 16.7% | 38.1% | 1.00 | Pending (carried to Round 3) | Carried to Round 3 |
| Perform pericardiocentesis | 2nd time | 2.5 | 4.8% | 64.3% | 1.50 | Non-adopted (persistent lack of consensus) | — |
| Insert an intraosseous needle | 2nd time | 6 | 59.5% | 9.5% | 0.50 | Non-adopted (persistent lack of consensus) | — |
| Insert an arterial line | 2nd time | 5 | 47.6% | 14.3% | 0.88 | Non-adopted (persistent lack of consensus) | — |
| Perform vagal manoeuvres (e.g., Valsalva manoeuvre) | 2nd time | 6 | 73.8% | 0.0% | 0.88 | Non-adopted (persistent lack of consensus) | — |
| Assess the indication for vasopressor | 1st time | 6 | 90.5% | 0.0% | 0.50 | Adopted | — |
| Manage circulation with vasopressors until handover to a specialist | 1st time | 6 | 90.5% | 0.0% | 0.50 | Adopted | — |
| Assess and reduce benign paroxysmal positional vertigo (e.g., Dix–Hallpike test, supine roll test, Epley, Gufoni, and Barbecue rotation manoeuvres) | 2nd time | 6 | 52.4% | 4.8% | 0.50 | Non-adopted (persistent lack of consensus) | — |
| Assess consciousness using GCS/JCS | 1st time | 7 | 97.6% | 0.0% | 0.00 | Adopted | — |
| Assess stroke severity using the NIHSS | 1st time | 6 | 59.5% | 14.3% | 0.50 | Pending (carried to Round 3) | Carried to Round 3 |
| Apply a tourniquet for haemorrhage control | 2nd time | 5 | 45.2% | 16.7% | 0.50 | Non-adopted (persistent lack of consensus) | — |
| Apply a pelvic binder | 2nd time | 5 | 28.6% | 19.0% | 1.00 | Non-adopted (persistent lack of consensus) | — |
| Reduce a shoulder dislocation | 2nd time | 5 | 23.8% | 31.0% | 1.00 | Non-adopted (persistent lack of consensus) | — |
| Reduce nursemaid's elbow | 2nd time | 6 | 76.2% | 2.4% | 0.50 | Non-adopted (persistent lack of consensus) | — |
| Reduce a temporomandibular joint dislocation | 2nd time | 6 | 61.9% | 4.8% | 0.50 | Non-adopted (persistent lack of consensus) | — |
| Reduce distal radius and ulna fractures | 2nd time | 4 | 2.4% | 45.2% | 1.50 | Non-adopted (persistent lack of consensus) | — |
| Apply a splint to the upper extremity | 2nd time | 6 | 71.4% | 0.0% | 0.50 | Non-adopted (persistent lack of consensus) | — |
| Apply a splint to the lower extremity | 2nd time | 6 | 71.4% | 0.0% | 0.50 | Non-adopted (persistent lack of consensus) | — |
| Apply traction to the lower extremity | 2nd time | 2.5 | 2.4% | 71.4% | 1.00 | Non-adopted (consensus) | — |
| Apply a cervical collar | 2nd time | 6 | 71.4% | 2.4% | 1.00 | Non-adopted (persistent lack of consensus) | — |
| Apply a backboard | 2nd time | 6 | 59.5% | 9.5% | 0.50 | Non-adopted (persistent lack of consensus) | — |
| Perform a foreign body removal with simple incision | 2nd time | 6 | 54.8% | 7.1% | 0.50 | Non-adopted (persistent lack of consensus) | — |
| Assess burns (depth and extent) | 1st time | 6 | 59.5% | 11.9% | 0.88 | Pending (carried to Round 3) | Carried to Round 3 |
| Provide conservative management (topical agents and dressings) for mild-to-moderate burns (up to superficial partial-thickness) | 1st time | 6 | 64.3% | 2.4% | 0.50 | Pending (carried to Round 3) | Carried to Round 3 |
| Measure intra-abdominal (bladder) pressure | 2nd time | 3 | 9.5% | 61.9% | 1.00 | Non-adopted (persistent lack of consensus) | — |
| Measure intracompartmental pressure | 2nd time | 2 | 2.4% | 78.6% | 0.50 | Non-adopted (consensus) | — |
| Interpret extremity radiographs | 2nd time | 6 | 78.6% | 2.4% | 0.38 | Non-adopted (persistent lack of consensus) | — |
| Interpret spinal CT scans | 2nd time | 6 | 64.3% | 4.8% | 0.50 | Non-adopted (persistent lack of consensus) | — |
| Interpret extremity CT scans | 2nd time | 6 | 64.3% | 4.8% | 0.50 | Non-adopted (persistent lack of consensus) | — |
| Interpret brain MRI | 2nd time | 6 | 78.6% | 0.0% | 0.38 | Non-adopted (persistent lack of consensus) | — |
| Interpret spinal MRI | 2nd time | 6 | 57.1% | 14.3% | 0.50 | Non-adopted (persistent lack of consensus) | — |
| Perform cardiac POCUS | 2nd time | 6 | 66.7% | 2.4% | 0.88 | Non-adopted (persistent lack of consensus) | — |
| Perform lung POCUS | 2nd time | 5 | 45.2% | 19.0% | 1.00 | Non-adopted (persistent lack of consensus) | — |
| Perform airway POCUS | 2nd time | 4 | 21.4% | 40.5% | 1.00 | Non-adopted (persistent lack of consensus) | — |
| Perform abdominal POCUS | 2nd time | 6 | 73.8% | 4.8% | 0.88 | Non-adopted (persistent lack of consensus) | — |
| Perform POCUS for deep vein thrombosis | 2nd time | 5 | 47.6% | 7.1% | 0.50 | Non-adopted (persistent lack of consensus) | — |
| Perform obstetric and gynaecologic POCUS | 1st time | 3 | 7.1% | 54.8% | 1.38 | Pending (carried to Round 3) | Carried to Round 3 |
| Perform urologic POCUS | 1st time | 5 | 21.4% | 31.0% | 1.00 | Pending (carried to Round 3) | Carried to Round 3 |
| Perform carotid artery POCUS | 1st time | 5 | 23.8% | 26.2% | 0.88 | Pending (carried to Round 3) | Carried to Round 3 |
| Perform anoscopy | 2nd time | 5 | 45.2% | 19.0% | 1.00 | Non-adopted (persistent lack of consensus) | — |
| Perform gastric lavage | 2nd time | 5 | 31.0% | 35.7% | 1.50 | Non-adopted (persistent lack of consensus) | — |
| Insert an intestinal decompression tube under fluoroscopic guidance | 1st time | 2 | 2.4% | 78.6% | 0.50 | Non-adopted (consensus) | — |
| Insert a Sengstaken–Blakemore tube | 2nd time | 2 | 9.5% | 69.0% | 1.00 | Non-adopted (persistent lack of consensus) | — |
| Perform paracentesis | 2nd time | 6 | 76.2% | 9.5% | 0.50 | Non-adopted (persistent lack of consensus) | — |
| Assess the indication for emergency haemodialysis | 2nd time | 6 | 90.5% | 0.0% | 0.50 | Adopted | — |
| Perform emergency haemodialysis | 2nd time | 3.5 | 11.9% | 50.0% | 1.50 | Non-adopted (persistent lack of consensus) | — |
| Insert a dialysis catheter | 2nd time | 5 | 33.3% | 38.1% | 1.50 | Non-adopted (persistent lack of consensus) | — |
| Insert an indwelling urinary catheter for urinary retention (standard technique) | 1st time | 7 | 97.6% | 0.0% | 0.50 | Adopted | — |
| Insert an indwelling urinary catheter for urinary retention (using a guidewire) | 1st time | 3 | 11.9% | 57.1% | 1.50 | Pending (carried to Round 3) | Carried to Round 3 |
| Provide initial neonatal care after precipitous delivery | 1st time | 2 | 11.9% | 69.0% | 1.38 | Pending (carried to Round 3) | Carried to Round 3 |
| Perform procedural sedation and analgesia | 2nd time | 6 | 78.6% | 0.0% | 0.50 | Non-adopted (persistent lack of consensus) | — |
| Perform a digital nerve block | 2nd time | 6 | 54.8% | 4.8% | 0.50 | Non-adopted (persistent lack of consensus) | — |
| Perform regional anaesthesia other than digital block | 2nd time | 2.5 | 4.8% | 61.9% | 1.00 | Non-adopted (persistent lack of consensus) | — |
| Perform ocular irrigation | 2nd time | 6 | 76.2% | 2.4% | 0.00 | Non-adopted (persistent lack of consensus) | — |
| Perform slit-lamp examination of the cornea | 2nd time | 2 | 0.0% | 76.2% | 0.50 | Non-adopted (consensus) | — |
| Remove a corneal foreign body | 2nd time | 3 | 11.9% | 52.4% | 1.50 | Non-adopted (persistent lack of consensus) | — |
| Assess nystagmus using Frenzel goggles | 2nd time | 5 | 45.2% | 11.9% | 0.50 | Non-adopted (persistent lack of consensus) | — |
| Remove a foreign body from the external auditory canal | 2nd time | 5 | 47.6% | 7.1% | 0.50 | Non-adopted (persistent lack of consensus) | — |
| Perform anterior nasal packing | 2nd time | 6 | 76.2% | 0.0% | 0.00 | Non-adopted (persistent lack of consensus) | — |
| Perform posterior nasal packing using a balloon catheter | 2nd time | 3 | 9.5% | 54.8% | 1.50 | Non-adopted (persistent lack of consensus) | — |
| Perform fibreoptic laryngoscopy for airway evaluation | 2nd time | 3 | 9.5% | 61.9% | 1.50 | Non-adopted (persistent lack of consensus) | — |
| Remove a pharyngeal or laryngeal foreign body | 2nd time | 5 | 16.7% | 33.3% | 1.00 | Non-adopted (persistent lack of consensus) | — |
| Perform incision and drainage of an abscess | 2nd time | 6 | 57.1% | 11.9% | 0.88 | Non-adopted (persistent lack of consensus) | — |
| Perform debridement of pressure ulcers | 2nd time | 5 | 42.9% | 26.2% | 1.38 | Non-adopted (persistent lack of consensus) | — |
| Perform Gram staining of prepared specimens | 2nd time | 6 | 57.1% | 9.5% | 0.88 | Non-adopted (persistent lack of consensus) | — |
| Perform paediatric advanced life support (PALS) | 2nd time | 6 | 52.4% | 16.7% | 0.88 | Non-adopted (persistent lack of consensus) | — |
| Perform standardised initial trauma care (JATEC) | 2nd time | 6 | 61.9% | 4.8% | 0.50 | Non-adopted (persistent lack of consensus) | — |
| Perform obstetric emergency care (ALSO/BLSO) | 2nd time | 4 | 19.0% | 33.3% | 1.00 | Non-adopted (persistent lack of consensus) | — |
| Perform surface cooling for heat stroke (e.g., ice packs, evaporative cooling with mist and fans) | 1st time | 7 | 92.9% | 0.0% | 0.50 | Adopted | — |
| Perform cold-water immersion (ice bath) for heat stroke | 1st time | 5 | 35.7% | 31.0% | 1.50 | Pending (carried to Round 3) | Carried to Round 3 |
| Perform invasive cooling for heat stroke (e.g., intracorporeal, intravascular, or extracorporeal cooling) | 1st time | 3 | 11.9% | 57.1% | 1.00 | Pending (carried to Round 3) | Carried to Round 3 |
| Perform non-invasive rewarming for hypothermia (e.g., warming blankets, forced-air warmers, warmed intravenous fluids) | 1st time | 7 | 95.2% | 0.0% | 0.50 | Adopted | — |
| Perform minimally invasive rewarming for hypothermia (e.g., gastric or thoracic lavage) | 1st time | 3 | 4.8% | 69.0% | 1.00 | Pending (carried to Round 3) | Carried to Round 3 |
| Perform invasive rewarming for hypothermia (extracorporeal circulation) | 1st time | 2 | 2.4% | 85.7% | 0.50 | Non-adopted (consensus) | — |
| Assess the indication for hyperbaric oxygen therapy | 2nd time | 3 | 11.9% | 52.4% | 1.50 | Non-adopted (persistent lack of consensus) | — |
| Identify toxic substances based on toxidromes | 2nd time | 5 | 16.7% | 26.2% | 0.88 | Non-adopted (persistent lack of consensus) | — |
| Provide initial treatment for poisoning based on the substance (supportive care and assessment of indication for specific therapy) | 1st time | 5 | 28.6% | 23.8% | 1.00 | Pending (carried to Round 3) | Carried to Round 3 |
| Apply PPE for CBRN incidents | 2nd time | 4 | 14.3% | 42.9% | 1.50 | Non-adopted (persistent lack of consensus) | — |
| Perform primary triage using the START system | 2nd time | 6 | 66.7% | 2.4% | 1.00 | Non-adopted (persistent lack of consensus) | — |
| Perform secondary triage using the PAT system | 2nd time | 6 | 54.8% | 7.1% | 0.88 | Non-adopted (persistent lack of consensus) | — |
| Perform and assess neurological examinations for brain death determination | 2nd time | 2 | 2.4% | 76.2% | 0.50 | Non-adopted (consensus) | — |
| Provide online medical direction to paramedics | 2nd time | 5 | 26.2% | 26.2% | 1.25 | Non-adopted (persistent lack of consensus) | — |

**Legend and summary**

| **Round count** | |
| --- | --- |
| **1st time** | Newly introduced in Round 2 (split from Round 1 items or added based on Round 1 panel feedback). |
| **2nd time** | Carried over from Round 1 without consensus. |
| **Consensus category** | |
| **Adopted** | ≥80% scoring 6–7, median ≥6, quartile deviation ≤1 |
| **Non-adopted (consensus)** | ≥70% scoring 1–3, median ≤3, quartile deviation ≤2 |
| **Non-adopted (persistent lack of consensus)** | Failed to reach adoption or consensus non-adoption in two consecutive rounds. |
| **Pending (carried to Round 3)** | No consensus reached in this round; carried over to Round 3. |
| **Round 2 summary** | |
| **Total items evaluated** | 108 (39 newly introduced + 69 carried over) |
| **Adopted** | 15 |
| **Non-adopted (consensus)** | 6 |
| **Non-adopted (persistent lack of consensus)** | 62 |
| **Pending (carried to Round 3)** | 25 |
| **Response rate** | 42 / 48 (87.5%) |
