# Supplementary material for: Emergency care competencies for non–emergency medicine specialists in Japan: a modified Delphi study
Source: BMC Med Educ. 2026 Aug 8;26:1786. doi: 10.1186/s12909-026-10094-0 (PMC13732881; doi:10.1186/s12909-026-10094-0)
Supplement: Supplementary file 5 [file 12909_2026_10094_MOESM5_ESM.docx]

**Supplemental Table S5. Round 3 results**

| **Item** | **Round count** | **Median** | **% rated 6–7** | **% rated 1–3** | **QD** | **Consensus category** | **Next round** |
| --- | --- | --- | --- | --- | --- | --- | --- |
| Assess anatomically difficult airways (e.g., using the LEMON criteria) | 2nd time | 6 | 58.5% | 7.3% | 0.50 | Non-adopted (persistent lack of consensus) | — |
| Assess physiologically difficult airways (e.g., hypoxia, hypotension, metabolic acidosis) | 2nd time | 6 | 73.2% | 2.4% | 0.50 | Non-adopted (persistent lack of consensus) | — |
| Assess the indication for neuromuscular blockade in non-cardiac-arrest patients | 2nd time | 5 | 26.8% | 24.4% | 1.00 | Non-adopted (persistent lack of consensus) | — |
| Perform orotracheal intubation in non-cardiac-arrest patients without anatomical difficulty using a direct laryngoscope | 2nd time | 6 | 70.7% | 9.8% | 1.00 | Non-adopted (persistent lack of consensus) | — |
| Perform orotracheal intubation in non-cardiac-arrest patients without anatomical difficulty using a video laryngoscope | 2nd time | 6 | 75.6% | 4.9% | 0.50 | Non-adopted (persistent lack of consensus) | — |
| Perform orotracheal intubation in non-cardiac-arrest patients with anatomically difficult airways using a video laryngoscope | 2nd time | 4 | 22.0% | 41.5% | 1.00 | Non-adopted (persistent lack of consensus) | — |
| Use intubation adjunct devices (e.g., gum elastic bougie) for difficult intubation | 2nd time | 4 | 9.8% | 43.9% | 1.00 | Non-adopted (persistent lack of consensus) | — |
| Insert a supraglottic airway device in cardiac arrest patients | 2nd time | 6 | 56.1% | 7.3% | 1.00 | Non-adopted (persistent lack of consensus) | — |
| Insert a supraglottic airway device in non-cardiac-arrest patients | 2nd time | 5 | 36.6% | 17.1% | 1.00 | Non-adopted (persistent lack of consensus) | — |
| Perform airway foreign body removal using Magill forceps under laryngoscopy | 2nd time | 6 | 63.4% | 2.4% | 0.50 | Non-adopted (persistent lack of consensus) | — |
| Manage mechanical ventilation until handover to a specialist | 2nd time | 6 | 75.6% | 2.4% | 0.50 | Non-adopted (persistent lack of consensus) | — |
| Perform needle thoracentesis for tension pneumothorax | 2nd time | 6 | 78.0% | 0.0% | 0.00 | Non-adopted (persistent lack of consensus) | — |
| Manage a pacemaker device (in collaboration with a clinical engineer) | 2nd time | 4 | 9.8% | 46.3% | 1.50 | Non-adopted (persistent lack of consensus) | — |
| Assess stroke severity using the NIHSS | 2nd time | 6 | 53.7% | 7.3% | 0.50 | Non-adopted (persistent lack of consensus) | — |
| Assess burns (depth and extent) | 2nd time | 6 | 51.2% | 7.3% | 0.50 | Non-adopted (persistent lack of consensus) | — |
| Provide conservative management (topical agents and dressings) for mild-to-moderate burns (up to superficial partial-thickness) | 2nd time | 6 | 70.7% | 4.9% | 0.50 | Non-adopted (persistent lack of consensus) | — |
| Perform obstetric and gynaecologic POCUS | 2nd time | 3 | 9.8% | 58.5% | 1.00 | Non-adopted (persistent lack of consensus) | — |
| Perform urologic POCUS | 2nd time | 5 | 22.0% | 19.5% | 0.50 | Non-adopted (persistent lack of consensus) | — |
| Perform carotid artery POCUS | 2nd time | 5 | 22.0% | 29.3% | 1.00 | Non-adopted (persistent lack of consensus) | — |
| Insert an indwelling urinary catheter for urinary retention (using a guidewire) | 2nd time | 3 | 14.6% | 58.5% | 1.50 | Non-adopted (persistent lack of consensus) | — |
| Provide initial neonatal care after precipitous delivery | 2nd time | 2 | 4.9% | 75.6% | 0.50 | Non-adopted (consensus) | — |
| Perform cold-water immersion (ice bath) for heat stroke | 2nd time | 4 | 31.7% | 36.6% | 2.00 | Non-adopted (persistent lack of consensus) | — |
| Perform invasive cooling for heat stroke (e.g., intracorporeal, intravascular, or extracorporeal cooling) | 2nd time | 2 | 9.8% | 73.2% | 1.00 | Non-adopted (consensus) | — |
| Perform minimally invasive rewarming for hypothermia (e.g., gastric or thoracic lavage) | 2nd time | 2 | 2.4% | 82.9% | 0.50 | Non-adopted (consensus) | — |
| Provide initial treatment for poisoning based on the substance (supportive care and assessment of indication for specific therapy) | 2nd time | 5 | 22.0% | 22.0% | 0.50 | Non-adopted (persistent lack of consensus) | — |

**Legend and summary**

| **Round count** | |
| --- | --- |
| **2nd time** | All Round 3 items were evaluated for the second time, having been carried over from Round 2 without consensus. |
| **Consensus category** | |
| **Adopted** | ≥80% scoring 6–7, median ≥6, quartile deviation ≤1 |
| **Non-adopted (consensus)** | ≥70% scoring 1–3, median ≤3, quartile deviation ≤2 |
| **Non-adopted (persistent lack of consensus)** | Failed to reach adoption or consensus non-adoption in two consecutive rounds. |
| **Round 3 summary (final round)** | |
| **Total items evaluated** | 25 (all carried over from Round 2) |
| **Adopted** | 0 |
| **Non-adopted (consensus)** | 3 |
| **Non-adopted (persistent lack of consensus)** | 22 |
| **Response rate** | 41 / 42 (97.6%) |
| **Final cumulative results across all three rounds** | |
| **Adopted (total)** | 38 (Round 1: 23 + Round 2: 15 + Round 3: 0) |
| **Non-adopted (total)** | 109 |
| **Non-adopted (consensus)** | 25 (Round 1: 16 + Round 2: 6 + Round 3: 3) |
| **Non-adopted (persistent lack of consensus)** | 84 (Round 2: 62 + Round 3: 22) |
