# Supplementary material for: Emergency care competencies for non–emergency medicine specialists in Japan: a modified Delphi study
Source: BMC Med Educ. 2026 Aug 8;26:1786. doi: 10.1186/s12909-026-10094-0 (PMC13732881; doi:10.1186/s12909-026-10094-0)
Supplement: Supplementary file 6 [file 12909_2026_10094_MOESM6_ESM.docx]

**Supplementary Table S6. Subgroup medians of the 38 adopted competency items by expertise group.**

| **Item** | **Median** | |
| --- | --- | --- |
|  | **EM** | **non-EM** |
| **Airway** | | |
| Insert an oropharyngeal airway | 7.0 | 7.0 |
| Insert a nasopharyngeal airway | 7.0 | 7.0 |
| Perform orotracheal intubation in cardiac arrest patients using a direct laryngoscope | 6.0 | 7.0 |
| Perform orotracheal intubation in cardiac arrest patients using a video laryngoscope | 6.5 | 6.5 |
| Perform airway foreign body removal using back blows and the Heimlich manoeuvre | 6.0 | 7.0 |
| Perform tracheostomy tube replacement | 6.0 | 6.0 |
| **Breathing** | | |
| Perform bag-valve-mask ventilation | 7.0 | 7.0 |
| Administer high-flow nasal oxygen therapy | 6.0 | 6.0 |
| Perform non-invasive positive pressure ventilation | 6.0 | 6.0 |
| **Circulation** | | |
| Insert a peripheral intravenous line | 7.0 | 7.0 |
| Insert a central venous catheter | 6.5 | 7.0 |
| Manage circulation with vasopressors until handover to a specialist | 6.0 | 6.5 |
| **Neurological assessment** | | |
| Assess consciousness using GCS/JCS | 7.0 | 7.0 |
| **Testing procedure and interpretation** | | |
| Perform lumbar puncture | 6.5 | 7.0 |
| Interpret electrocardiograms | 6.5 | 7.0 |
| Perform arterial blood sampling | 7.0 | 7.0 |
| Interpret blood gas analysis | 7.0 | 7.0 |
| Interpret routine blood tests | 7.0 | 7.0 |
| Interpret cerebrospinal fluid analysis | 7.0 | 7.0 |
| Perform diagnostic thoracentesis for pleural effusion | 6.0 | 6.0 |
| **Imaging interpretation** | | |
| Interpret chest radiographs | 7.0 | 7.0 |
| Interpret head CT scans | 6.0 | 7.0 |
| Interpret chest CT scans | 6.0 | 7.0 |
| Interpret abdominal CT scans | 6.0 | 7.0 |
| Perform FAST | 7.0 | 7.0 |
| **Resuscitation procedures** | | |
| Perform basic life support (BLS) | 7.0 | 7.0 |
| Perform advanced cardiovascular life support (ACLS) | 7.0 | 7.0 |
| **Indication judgement** | | |
| Assess the indication for mechanical ventilation | 6.0 | 7.0 |
| Assess the indication for vasopressor | 6.0 | 6.5 |
| Assess the indication for emergency haemodialysis | 6.0 | 6.5 |
| **Bedside procedures** | | |
| Perform direct compression for haemorrhage control | 7.0 | 7.0 |
| Perform simple wound suturing | 7.0 | 7.0 |
| Insert a nasogastric tube | 7.0 | 7.0 |
| Insert an indwelling urinary catheter for urinary retention (standard technique) | 7.0 | 7.0 |
| Perform surface cooling for heat stroke (e.g., ice packs, evaporative cooling with mist and fans) | 6.0 | 7.0 |
| Perform non-invasive rewarming for hypothermia (e.g., warming blankets, forced-air warmers, warmed intravenous fluids) | 6.5 | 7.0 |
| **Infection control** | | |
| Perform appropriate donning and doffing of personal protective equipment | 7.0 | 7.0 |
| **Legal documentation** | | |
| Complete death certificates and postmortem examination reports | 7.0 | 7.0 |

EM, emergency medicine; non-EM, non-emergency medicine. Values are within-subgroup medians at the round of adoption. All 38 items had a median of ≥6 in both subgroups.
