# Supplementary material for: Emergency care competencies for non–emergency medicine specialists in Japan: a modified Delphi study
Source: BMC Med Educ. 2026 Aug 8;26:1786. doi: 10.1186/s12909-026-10094-0 (PMC13732881; doi:10.1186/s12909-026-10094-0)
Supplement: Supplementary file 7 [file 12909_2026_10094_MOESM7_ESM.docx]

**Supplementary Table S7. Non-adopted competency items, with non-adoption pathway and round of classification.**

| **Item** | **Round non-adopted** | **Reason for non-adoption** | **Median** | **Median (EM)** | **Median (non-EM)** | **% rated 6–7** | **% rated 1–3** | **QD** |
| --- | --- | --- | --- | --- | --- | --- | --- | --- |
| Perform fibreoptic nasotracheal intubation | R1 | Consensus non-adoption | 2.0 | 2.0 | 3.0 | 6.3% | 72.9% | 1.50 |
| Perform tracheostomy | R1 | Consensus non-adoption | 1.0 | 1.0 | 2.0 | 0.0% | 91.7% | 0.50 |
| Perform thoracotomy | R1 | Consensus non-adoption | 1.0 | 1.0 | 1.0 | 0.0% | 97.9% | 0.00 |
| Perform open-chest cardiac massage | R1 | Consensus non-adoption | 1.0 | 1.0 | 1.0 | 0.0% | 100.0% | 0.00 |
| Perform transvenous pacing | R1 | Consensus non-adoption | 1.0 | 1.0 | 2.0 | 0.0% | 95.8% | 0.50 |
| Perform pericardial window | R1 | Consensus non-adoption | 1.0 | 1.0 | 1.0 | 0.0% | 100.0% | 0.50 |
| Insert a pulmonary artery catheter | R1 | Consensus non-adoption | 1.0 | 1.0 | 2.0 | 0.0% | 89.6% | 1.00 |
| Initiate and manage intra-aortic balloon pumping | R1 | Consensus non-adoption | 1.0 | 1.0 | 1.0 | 0.0% | 95.8% | 0.50 |
| Initiate and manage extracorporeal membrane oxygenation | R1 | Consensus non-adoption | 1.0 | 1.0 | 1.0 | 0.0% | 97.9% | 0.13 |
| Insert a REBOA (resuscitative endovascular balloon occlusion of the aorta) catheter | R1 | Consensus non-adoption | 1.0 | 1.0 | 1.0 | 0.0% | 91.7% | 0.50 |
| Insert an intracranial pressure monitor | R1 | Consensus non-adoption | 1.0 | 1.0 | 2.0 | 0.0% | 93.8% | 0.50 |
| Reduce a hip dislocation | R1 | Consensus non-adoption | 3.0 | 2.0 | 3.0 | 4.2% | 72.9% | 1.00 |
| Perform escharotomy | R1 | Consensus non-adoption | 2.0 | 1.0 | 3.0 | 0.0% | 81.3% | 1.00 |
| Perform suprapubic bladder puncture | R1 | Consensus non-adoption | 3.0 | 2.0 | 3.0 | 4.2% | 75.0% | 0.63 |
| Assist vaginal delivery | R1 | Consensus non-adoption | 2.0 | 2.0 | 3.0 | 2.1% | 70.8% | 1.00 |
| Perform lateral canthotomy | R1 | Consensus non-adoption | 1.0 | 1.0 | 1.5 | 2.1% | 93.8% | 0.50 |
| Perform needle cricothyrotomy | R2 | Persistent lack of consensus | 4.0 | 3.0 | 5.0 | 19.0% | 45.2% | 1.38 |
| Perform cricothyrotomy | R2 | Persistent lack of consensus | 3.0 | 3.0 | 3.0 | 11.9% | 64.3% | 1.00 |
| Insert a chest tube | R2 | Persistent lack of consensus | 5.0 | 5.0 | 5.0 | 38.1% | 19.0% | 0.88 |
| Perform bronchoscopic airway suctioning and assessment | R2 | Persistent lack of consensus | 3.0 | 3.0 | 3.0 | 7.1% | 57.1% | 1.50 |
| Perform synchronised cardioversion for arrhythmias | R2 | Persistent lack of consensus | 6.0 | 6.0 | 6.0 | 54.8% | 7.1% | 0.50 |
| Perform transcutaneous pacing | R2 | Persistent lack of consensus | 5.0 | 5.0 | 6.0 | 47.6% | 11.9% | 0.50 |
| Perform pericardiocentesis | R2 | Persistent lack of consensus | 2.5 | 2.5 | 2.5 | 4.8% | 64.3% | 1.50 |
| Insert an intraosseous needle | R2 | Persistent lack of consensus | 6.0 | 5.5 | 6.0 | 59.5% | 9.5% | 0.50 |
| Insert an arterial line | R2 | Persistent lack of consensus | 5.0 | 5.0 | 6.0 | 47.6% | 14.3% | 0.88 |
| Perform vagal manoeuvres (e.g., Valsalva manoeuvre) | R2 | Persistent lack of consensus | 6.0 | 6.0 | 6.0 | 73.8% | 0.0% | 0.88 |
| Assess and reduce benign paroxysmal positional vertigo (e.g., Dix–Hallpike test, supine roll test, Epley, Gufoni, and Barbecue rotation manoeuvres) | R2 | Persistent lack of consensus | 6.0 | 5.5 | 6.0 | 52.4% | 4.8% | 0.50 |
| Apply a tourniquet for haemorrhage control | R2 | Persistent lack of consensus | 5.0 | 5.0 | 5.5 | 45.2% | 16.7% | 0.50 |
| Apply a pelvic binder | R2 | Persistent lack of consensus | 5.0 | 5.0 | 5.0 | 28.6% | 19.0% | 1.00 |
| Reduce a shoulder dislocation | R2 | Persistent lack of consensus | 5.0 | 5.0 | 4.0 | 23.8% | 31.0% | 1.00 |
| Reduce nursemaid's elbow | R2 | Persistent lack of consensus | 6.0 | 6.0 | 6.0 | 76.2% | 2.4% | 0.50 |
| Reduce a temporomandibular joint dislocation | R2 | Persistent lack of consensus | 6.0 | 5.5 | 6.0 | 61.9% | 4.8% | 0.50 |
| Reduce distal radius and ulna fractures | R2 | Persistent lack of consensus | 4.0 | 4.0 | 3.5 | 2.4% | 45.2% | 1.50 |
| Apply a splint to the upper extremity | R2 | Persistent lack of consensus | 6.0 | 6.0 | 6.0 | 71.4% | 0.0% | 0.50 |
| Apply a splint to the lower extremity | R2 | Persistent lack of consensus | 6.0 | 6.0 | 6.0 | 71.4% | 0.0% | 0.50 |
| Apply traction to the lower extremity | R2 | Consensus non-adoption | 2.5 | 2.0 | 3.0 | 2.4% | 71.4% | 1.00 |
| Apply a cervical collar | R2 | Persistent lack of consensus | 6.0 | 6.0 | 6.0 | 71.4% | 2.4% | 1.00 |
| Apply a backboard | R2 | Persistent lack of consensus | 6.0 | 5.0 | 6.0 | 59.5% | 9.5% | 0.50 |
| Perform a foreign body removal with simple incision | R2 | Persistent lack of consensus | 6.0 | 5.5 | 6.0 | 54.8% | 7.1% | 0.50 |
| Measure intra-abdominal (bladder) pressure | R2 | Persistent lack of consensus | 3.0 | 3.5 | 2.5 | 9.5% | 61.9% | 1.00 |
| Measure intracompartmental pressure | R2 | Consensus non-adoption | 2.0 | 2.5 | 2.0 | 2.4% | 78.6% | 0.50 |
| Interpret extremity radiographs | R2 | Persistent lack of consensus | 6.0 | 6.0 | 6.0 | 78.6% | 2.4% | 0.38 |
| Interpret spinal CT scans | R2 | Persistent lack of consensus | 6.0 | 6.0 | 6.0 | 64.3% | 4.8% | 0.50 |
| Interpret extremity CT scans | R2 | Persistent lack of consensus | 6.0 | 6.0 | 6.0 | 64.3% | 4.8% | 0.50 |
| Interpret brain MRI | R2 | Persistent lack of consensus | 6.0 | 6.0 | 6.0 | 78.6% | 0.0% | 0.38 |
| Interpret spinal MRI | R2 | Persistent lack of consensus | 6.0 | 5.5 | 6.0 | 57.1% | 14.3% | 0.50 |
| Perform cardiac POCUS | R2 | Persistent lack of consensus | 6.0 | 6.0 | 6.0 | 66.7% | 2.4% | 0.88 |
| Perform lung POCUS | R2 | Persistent lack of consensus | 5.0 | 5.0 | 5.5 | 45.2% | 19.0% | 1.00 |
| Perform airway POCUS | R2 | Persistent lack of consensus | 4.0 | 4.0 | 4.0 | 21.4% | 40.5% | 1.00 |
| Perform abdominal POCUS | R2 | Persistent lack of consensus | 6.0 | 6.0 | 6.0 | 73.8% | 4.8% | 0.88 |
| Perform POCUS for deep vein thrombosis | R2 | Persistent lack of consensus | 5.0 | 5.0 | 6.0 | 47.6% | 7.1% | 0.50 |
| Perform anoscopy | R2 | Persistent lack of consensus | 5.0 | 5.5 | 5.0 | 45.2% | 19.0% | 1.00 |
| Perform gastric lavage | R2 | Persistent lack of consensus | 5.0 | 3.5 | 5.0 | 31.0% | 35.7% | 1.50 |
| Insert an intestinal decompression tube under fluoroscopic guidance | R2 | Consensus non-adoption | 2.0 | 2.0 | 2.0 | 2.4% | 78.6% | 0.50 |
| Insert a Sengstaken–Blakemore tube | R2 | Persistent lack of consensus | 2.0 | 2.0 | 2.0 | 9.5% | 69.0% | 1.00 |
| Perform paracentesis | R2 | Persistent lack of consensus | 6.0 | 6.0 | 6.0 | 76.2% | 9.5% | 0.50 |
| Perform emergency haemodialysis | R2 | Persistent lack of consensus | 3.5 | 3.0 | 4.0 | 11.9% | 50.0% | 1.50 |
| Insert a dialysis catheter | R2 | Persistent lack of consensus | 5.0 | 5.0 | 4.5 | 33.3% | 38.1% | 1.50 |
| Perform procedural sedation and analgesia | R2 | Persistent lack of consensus | 6.0 | 6.0 | 6.0 | 78.6% | 0.0% | 0.50 |
| Perform a digital nerve block | R2 | Persistent lack of consensus | 6.0 | 6.0 | 6.0 | 54.8% | 4.8% | 0.50 |
| Perform regional anaesthesia other than digital block | R2 | Persistent lack of consensus | 2.5 | 2.5 | 2.5 | 4.8% | 61.9% | 1.00 |
| Perform ocular irrigation | R2 | Persistent lack of consensus | 6.0 | 6.0 | 6.0 | 76.2% | 2.4% | 0.00 |
| Perform slit-lamp examination of the cornea | R2 | Consensus non-adoption | 2.0 | 3.0 | 2.0 | 0.0% | 76.2% | 0.50 |
| Remove a corneal foreign body | R2 | Persistent lack of consensus | 3.0 | 5.0 | 3.0 | 11.9% | 52.4% | 1.50 |
| Assess nystagmus using Frenzel goggles | R2 | Persistent lack of consensus | 5.0 | 5.0 | 5.0 | 45.2% | 11.9% | 0.50 |
| Remove a foreign body from the external auditory canal | R2 | Persistent lack of consensus | 5.0 | 5.0 | 6.0 | 47.6% | 7.1% | 0.50 |
| Perform anterior nasal packing | R2 | Persistent lack of consensus | 6.0 | 6.0 | 6.0 | 76.2% | 0.0% | 0.00 |
| Perform posterior nasal packing using a balloon catheter | R2 | Persistent lack of consensus | 3.0 | 3.5 | 3.0 | 9.5% | 54.8% | 1.50 |
| Perform fibreoptic laryngoscopy for airway evaluation | R2 | Persistent lack of consensus | 3.0 | 3.0 | 3.0 | 9.5% | 61.9% | 1.50 |
| Remove a pharyngeal or laryngeal foreign body | R2 | Persistent lack of consensus | 5.0 | 4.5 | 5.0 | 16.7% | 33.3% | 1.00 |
| Perform incision and drainage of an abscess | R2 | Persistent lack of consensus | 6.0 | 5.5 | 6.0 | 57.1% | 11.9% | 0.88 |
| Perform debridement of pressure ulcers | R2 | Persistent lack of consensus | 5.0 | 5.0 | 5.0 | 42.9% | 26.2% | 1.38 |
| Perform Gram staining of prepared specimens | R2 | Persistent lack of consensus | 6.0 | 5.5 | 6.0 | 57.1% | 9.5% | 0.88 |
| Perform paediatric advanced life support (PALS) | R2 | Persistent lack of consensus | 6.0 | 5.0 | 6.0 | 52.4% | 16.7% | 0.88 |
| Perform standardised initial trauma care (JATEC) | R2 | Persistent lack of consensus | 6.0 | 6.0 | 6.0 | 61.9% | 4.8% | 0.50 |
| Perform obstetric emergency care (ALSO/BLSO) | R2 | Persistent lack of consensus | 4.0 | 4.0 | 4.0 | 19.0% | 33.3% | 1.00 |
| Perform invasive rewarming for hypothermia (extracorporeal circulation) | R2 | Consensus non-adoption | 2.0 | 2.0 | 2.0 | 2.4% | 85.7% | 0.50 |
| Assess the indication for hyperbaric oxygen therapy | R2 | Persistent lack of consensus | 3.0 | 4.0 | 3.0 | 11.9% | 52.4% | 1.50 |
| Identify toxic substances based on toxidromes | R2 | Persistent lack of consensus | 5.0 | 5.0 | 5.0 | 16.7% | 26.2% | 0.88 |
| Apply PPE for CBRN incidents | R2 | Persistent lack of consensus | 4.0 | 4.0 | 4.0 | 14.3% | 42.9% | 1.50 |
| Perform primary triage using the START system | R2 | Persistent lack of consensus | 6.0 | 6.0 | 6.0 | 66.7% | 2.4% | 1.00 |
| Perform secondary triage using the PAT system | R2 | Persistent lack of consensus | 6.0 | 5.0 | 6.0 | 54.8% | 7.1% | 0.88 |
| Perform and assess neurological examinations for brain death determination | R2 | Consensus non-adoption | 2.0 | 2.0 | 2.0 | 2.4% | 76.2% | 0.50 |
| Provide online medical direction to paramedics | R2 | Persistent lack of consensus | 5.0 | 4.0 | 5.0 | 26.2% | 26.2% | 1.25 |
| Assess anatomically difficult airways (e.g., using the LEMON criteria) | R3 | Persistent lack of consensus | 6.0 | 6.0 | 5.5 | 58.5% | 7.3% | 0.50 |
| Assess physiologically difficult airways (e.g., hypoxia, hypotension, metabolic acidosis) | R3 | Persistent lack of consensus | 6.0 | 6.0 | 6.0 | 73.2% | 2.4% | 0.50 |
| Assess the indication for neuromuscular blockade in non-cardiac-arrest patients | R3 | Persistent lack of consensus | 5.0 | 5.0 | 5.0 | 26.8% | 24.4% | 1.00 |
| Perform orotracheal intubation in non-cardiac-arrest patients without anatomical difficulty using a direct laryngoscope | R3 | Persistent lack of consensus | 6.0 | 6.0 | 6.0 | 70.7% | 9.8% | 1.00 |
| Perform orotracheal intubation in non-cardiac-arrest patients without anatomical difficulty using a video laryngoscope | R3 | Persistent lack of consensus | 6.0 | 6.0 | 6.0 | 75.6% | 4.9% | 0.50 |
| Perform orotracheal intubation in non-cardiac-arrest patients with anatomically difficult airways using a video laryngoscope | R3 | Persistent lack of consensus | 4.0 | 3.0 | 5.0 | 22.0% | 41.5% | 1.00 |
| Use intubation adjunct devices (e.g., gum elastic bougie) for difficult intubation | R3 | Persistent lack of consensus | 4.0 | 4.0 | 4.0 | 9.8% | 43.9% | 1.00 |
| Insert a supraglottic airway device in cardiac arrest patients | R3 | Persistent lack of consensus | 6.0 | 6.0 | 6.0 | 56.1% | 7.3% | 1.00 |
| Insert a supraglottic airway device in non-cardiac-arrest patients | R3 | Persistent lack of consensus | 5.0 | 4.0 | 5.5 | 36.6% | 17.1% | 1.00 |
| Perform airway foreign body removal using Magill forceps under laryngoscopy | R3 | Persistent lack of consensus | 6.0 | 6.0 | 6.0 | 63.4% | 2.4% | 0.50 |
| Manage mechanical ventilation until handover to a specialist | R3 | Persistent lack of consensus | 6.0 | 6.0 | 6.5 | 75.6% | 2.4% | 0.50 |
| Perform needle thoracentesis for tension pneumothorax | R3 | Persistent lack of consensus | 6.0 | 6.0 | 6.0 | 78.0% | 0.0% | 0.00 |
| Manage a pacemaker device (in collaboration with a clinical engineer) | R3 | Persistent lack of consensus | 4.0 | 4.0 | 3.5 | 9.8% | 46.3% | 1.50 |
| Assess stroke severity using the NIHSS | R3 | Persistent lack of consensus | 6.0 | 5.0 | 6.0 | 53.7% | 7.3% | 0.50 |
| Assess burns (depth and extent) | R3 | Persistent lack of consensus | 6.0 | 5.0 | 6.0 | 51.2% | 7.3% | 0.50 |
| Provide conservative management (topical agents and dressings) for mild-to-moderate burns (up to superficial partial-thickness) | R3 | Persistent lack of consensus | 6.0 | 6.0 | 6.0 | 70.7% | 4.9% | 0.50 |
| Perform obstetric and gynaecologic POCUS | R3 | Persistent lack of consensus | 3.0 | 3.0 | 3.0 | 9.8% | 58.5% | 1.00 |
| Perform urologic POCUS | R3 | Persistent lack of consensus | 5.0 | 5.0 | 5.0 | 22.0% | 19.5% | 0.50 |
| Perform carotid artery POCUS | R3 | Persistent lack of consensus | 5.0 | 4.0 | 5.0 | 22.0% | 29.3% | 1.00 |
| Insert an indwelling urinary catheter for urinary retention (using a guidewire) | R3 | Persistent lack of consensus | 3.0 | 3.0 | 4.5 | 14.6% | 58.5% | 1.50 |
| Provide initial neonatal care after precipitous delivery | R3 | Consensus non-adoption | 2.0 | 2.0 | 2.0 | 4.9% | 75.6% | 0.50 |
| Perform cold-water immersion (ice bath) for heat stroke | R3 | Persistent lack of consensus | 4.0 | 4.0 | 5.0 | 31.7% | 36.6% | 2.00 |
| Perform invasive cooling for heat stroke (e.g., intracorporeal, intravascular, or extracorporeal cooling) | R3 | Consensus non-adoption | 2.0 | 2.0 | 3.0 | 9.8% | 73.2% | 1.00 |
| Perform minimally invasive rewarming for hypothermia (e.g., gastric or thoracic lavage) | R3 | Consensus non-adoption | 2.0 | 2.0 | 2.5 | 2.4% | 82.9% | 0.50 |
| Provide initial treatment for poisoning based on the substance (supportive care and assessment of indication for specific therapy) | R3 | Persistent lack of consensus | 5.0 | 5.0 | 4.5 | 22.0% | 22.0% | 0.50 |

EM, emergency medicine specialists; non-EM, non-emergency medicine specialists; QD, quartile deviation. Consensus non-adoption: ≥70% of ratings 1–3, median ≤3, QD ≤2. Persistent lack of consensus: no adoption or consensus non-adoption reached in two consecutive rounds.
